# Behavioural synchronisation between different groups of dogs and wolves and their owners/handlers: Exploring the effect of breed and human interaction

**DOI:** 10.1371/journal.pone.0302833

**Published:** 2024-05-03

**Authors:** Jasmine Heurlin, György Barabás, Lina S. V. Roth

**Affiliations:** 1 IFM Biology, Linköping University, Linköping, Sweden; 2 Institute of Evolution, HUN-REN Centre for Ecological Research, Konkoly-Thege Miklós út 29–33, Budapest, Hungary; Indian Institute of Science Education and Research Kolkata, INDIA

## Abstract

Dogs have previously been shown to synchronise their behaviour with their owner and the aim of this study was to test the effect of immediate interactions, breed, and the effects of domestication. The behavioural synchronisation test was conducted in outdoor enclosures and consisted of 30 s where the owner/handler was walking and 30 s of standing still. Three studies were conducted to explore the effect of immediate interaction (study A), the effect of breed group (study B), and the effect of domestication (study C). In study A, a group of twenty companion dogs of various breeds were tested after three different human interaction treatments: *Ignore*, *Pet*, and *Play*. The results showed that dogs adjusted their movement pattern to align with their owner’s actions regardless of treatment. Furthermore, exploration, eye contact, and movement were all influenced by the owners moving pattern, and exploration also decreased after the *Play* treatment. In study B, the synchronisation test was performed after the *Ignore* treatment on three groups: 24 dogs of ancient dog breeds, 17 solitary hunting dogs, and 20 companion dogs (data from study A). Irrespective of the group, all dogs synchronised their moving behaviour with their owner. In addition, human walking positively influenced eye contact behaviour while simultaneously decreasing exploration behaviour. In study C, a group of six socialised pack-living wolves and six similarly socialised pack-living dogs were tested after the *Ignore* treatment. Interestingly, these animals did not alter their moving behaviour in response to their handler. In conclusion, dogs living together with humans synchronise with their owner’s moving behaviour, while wolves and dogs living in packs do not. Hence, the degree of interspecies behavioural synchronisation may be influenced by the extent to which the dogs are immersed in everyday life with humans.

## Introduction

The domestic dog is well known for its abilities to communicate with humans [1] and in a pioneering study Miklósi et al. [2] compared dogs to similarly socialised wolves. They found that dogs show longer eye contact with humans than their ancestors, the wolves. Since then, there have been many comparative studies and also a recent review that challenges the current beliefs on socio-cognitive differences between dogs and wolves [3, but see also the response 4].

To further enhance our understanding of dog behaviour and the effect of the domestication process, it is important to investigate social cognition and contact-seeking behaviours in various situations. This is because some human-directed social skills in dogs may be unrelated to each other. For instance, dogs that seek a lot of human contact during a problem-solving task might be less sensitive to human ostensive cues [3]. The behaviour of dogs during problem-solving tasks has also been found to be influenced by breed and previous training [4,5]. On the other hand, the ability to interpret human pointing gestures is suggested to be more consistent throughout a dog’s life [e.g. 6; for review see 7]. Conducting tests where we do not actively challenge the animals with tasks or communicate directly with them can provide valuable additional insights. Nagasawa et al. [8] assessed the spontaneous contact-seeking behaviour in the presence of a passive human in a room, revealing once again that dogs maintain longer eye contact compared to wolves, even when the wolves are kept in pet-like conditions.

However, what would occur in a situation when individuals begin to move? It would be reasonable to hypothesise that domestication and modern breed selection have altered the animals’ urge to synchronise their behaviour with humans, but this has not been tested. For behaviour to be considered as behavioural synchronisation it should be similar and performed at a comparable pace, place and time [9,10]. Dogs have demonstrated consistent behavioural synchronisation, not only with other dogs, but also with adult humans both indoors and outdoors [11–13] and to a lesser extent, even with children [14]. Furthermore, it has been suggested that dogs synchronise more with individuals they have a closer relationship with [9,10,15]. Nonetheless, disentangling the effects of the domestication process from recent breed selection and life experiences [16,17] remains challenging. Investigating behavioural synchronisation in similarly socialised wolves and dogs together with their handlers, as well as in breeds selected for traits other than human cooperation and ancient dog breeds thought to be closer genetically to wolves, could enhance our understanding of the effects of the domestication process on human-dog behaviour and interspecies synchronisation.

Therefore, the objective of this study was to investigate the interspecies behavioural synchronisation of different groups of dogs and wolves together with their owners/handlers. All dyads underwent the same behavioural synchronisation test, and we also aimed to examine whether an owner’s interaction with the dog prior to the test could impact the synchronisation and/or contact-seeking behaviour. This could enhance our understanding of the evolutionary aspects of relationships between species, but it could also improve our ability to handle and train different types of dogs, as well as captive wolves. We hypothesised that regardless of breed, all dogs would exhibit some degree of synchronisation with their handler/owner, and that human interaction prior to the test would increase the duration of contact-seeking behaviour. Additionally, we hypothesised that dogs (and wolves) living solely with conspecifics would differ from companion dogs due to variations in the degree of the human-animal relationship and life experiences.

## Study A—the effect of human interaction

### Methods

#### Ethics declarations

**Fig 1:**
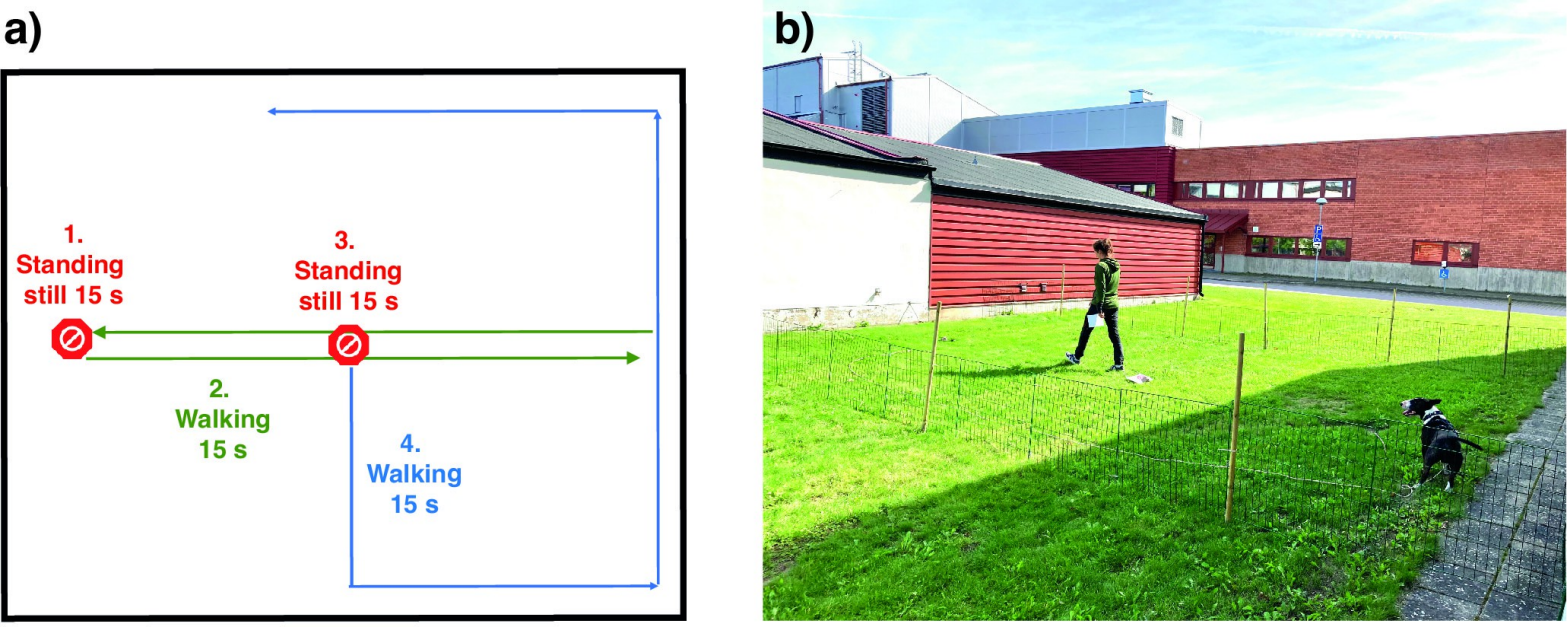
Experimental setting of the behavioural synchronization test. The behavioural synchronisation experiment included both a) standing still (phase 1 and 3) and walking phases (phases 2, indicated by green lines, and 4, indicated by blue lines) for the owner, and the experiment in study A and B was performed in an b) outdoor enclosure at Linköping University in the Southeast of Sweden.

In this study, we exclusively investigated the behaviour of privately owned dogs. No special ethical permission for use of privately owned dogs in non-invasive observational studies is required in Sweden (SJVFS 2019:9, 2 ch., 17§), nevertheless dog owners gave their written consent to voluntarily participate in the study which was executed in Linköping, Sweden. All methods were performed in accordance with the relevant guidelines and regulations and no animal displayed any signs of stress during the behavioural synchronisation. The sample size and animal information have been reported in accordance with ARRIVE guidelines (www.arriveguidelines.org). Additionally, consent to publish the photo in Fig 1 was obtained from the dog owner.

#### Subjects

Twenty dogs (11 females and 9 males) were recruited for the study through social media and personal contacts. All dogs were companion dogs living indoors and regularly walked, and they represented various breeds and breed groups. The ages of the dogs ranged from 1 to 9 years. For additional details about the dogs and their specific breeds, please see S1 Table. The experiments were conducted outdoors at Linköping University in the southeast of Sweden during April in 2019.

#### Experimental procedure

All dyads were individually tested in the following manner. Firstly, the owner was instructed to walk their dog on a loose leash in a clockwise direction inside the test arena. After completing one lap, they exit the test arena. Thereafter, the dyads performed the test procedure three times, with the owner receiving specific instructions before each trial. The instructions given were as follows: 1) *Ignore* the dog, 2) *Pet* the dog calmly or 3) *Play* with the dog for 1 min near the starting area. The owner was advised to maintain calm petting during the second instruction and to engage in play that resembled their typical play interaction with the dog at home. The order of treatment was balanced, and pseudo randomised between dyads with a 5-minute break between each test.

After one minute of interaction (or lack of interaction during the *Ignore* treatment), both the owner and the dog entered the starting area. At this point, the leash was removed from the dog and handed over to the test leader who stood next to the starting area. The owner was provided with a timer and a map. If the owner felt uncomfortable having the dog off-leash within the test arena, they were given the option to use a 10-meter-long leash, which was loose on the ground (no owner chose to use this leash). The test started when the owner reached the first designated standing still position (Fig 1; informed consent to publish Fig 1B was obtained from the dog owner).

The test procedure had a duration of one minute and remained consistent across all three treatments (Fig 1). The procedure was as follows: Firstly, the owner walked to the starting position and remained still for 15 s. Then, the owner was instructed to walk across the entire test arena to the opposite short side and back for 15 s. After that the owner took the shortest path to the middle of the arena, and stood still for an additional 15 s. Finally, the owner walked in a counter-clockwise direction along the fence/wall of the test arena for 15 s. Consequently, the owner spent a total of 30 s in the standing still phase and 30 s in the walking phase.

To assist the owners in remembering the test procedure, laminated paper signs were placed on the ground, and a small map (Fig 1) was provided to each owner. It was emphasised that the owner should refrain from interacting with their dogs during the actual test procedure. All dyads were video recorded for later analysis in Observer XT (Noldus) software, using a predetermined ethogram (Table 1).

**Table 1:** Ethogram of the analysed behaviours.

Term	Description	Source
Move	The animal is moving in any direction. Front paws are moving more than 5 cm.	Duranton et al. 2017
Same direction	The animal’s chest is pointed in the same direction as the humans’ hips, within 45° to either direction.	Wanser et al. 2021
Human proximity	The animal is within one dog length from the human.	Duranton et al. 2017
Eye contact	The animal’s nose is pointing towards the human’s head.	Duranton et al. 2017
Exploration	The animal has its nose within 10 cm from the ground or an object or licking/manipulating something.	
Out of view	If either the animal or human is out of view.	

### Data analysis

Our strategy for analysing the data is to break them up by behaviour and perform tests for each behaviour separately. While this precludes the study of interaction effects between behaviour type and either phase or treatment, these were not what we were interested in here, focusing instead on any possible interaction between the latter two (as well as their main effects). All calculations were done in R [18], version 4.2.2. Given the balanced and orthogonal design or our experiment (20 observations per every combination of phase and treatment, for each behaviour), the data appear ideally suited for performing a two-way ANOVA, using the equation

$${\left(\mathrm{fraction}\right)}_{i}=\beta_{0}+\beta_{1}{\left(\mathrm{phase}\right)}_{i}+\beta_{2}{\left(\mathrm{trt}\right)}_{i}+\beta_{3}{\left(\mathrm{phase}\right)}_{i}{\left(\mathrm{trt}\right)}_{i}+\epsilon_{i}$$ (1)

to predict the fraction of time spent on any one behaviour. Here “trt” is short for “treatment” (*Ignore/Pet/Play*), the *β*_*i*_ are standard regression coefficients, and *ε*_*i*_ is the residual for observation *i*. However, there are three important complicating factors to consider.

First, despite efforts, the duration of the walking phase and standing still phase could differ a few seconds between dyads. Therefore, all raw data were converted into a fraction of total time. Such a quantity is naturally confined to the [0, 1] range, and therefore the residual variation cannot strictly speaking be normally distributed. On the face of it, this means that one should use generalized linear models with a link function restricting the response to the [0, 1] interval. Second, the data are also zero-inflated, indicating behaviours that the dogs never exhibited during the experiment. Handling such data requires a mixture distribution in which a behaviour is not exhibited at all with some probability, and otherwise it follows a continuous distribution. And third, the data rely on repeated measurements, because each individual dog took part in all three treatments (*Ignore/Pet/Play*) in some order. Leaving uncontrolled (and uncontrollable) individual differences between dogs unaccounted for could artificially inflate the model’s confidence in its predictions. The way to handle this is to switch to mixed effect models where each dog may receive a random intercept:

$${\left(\mathrm{fraction}\right)}_{i}=\beta_{0}+\beta_{1}{\left(\mathrm{phase}\right)}_{i}+\beta_{2}{\left(\mathrm{trt}\right)}_{i}+\beta_{3}{\left(\mathrm{phase}\right)}_{i}{\left(\mathrm{trt}\right)}_{i}+(\mu_{id(i)}+\epsilon_{i}),$$ (2)

where id(*i*) is the identity of the dog in observation *i*, and *μ*_id(*i*)_ is the associated random intercept.

Fitting generalized linear models to deal with the problems of [0, 1] confinement and zero inflation in the response turns out not to be important, however. As shown in Section 1 in S1 File, not only are the assumptions behind Gaussian regression models reasonably satisfied, but other models (such as zero-inflated Gamma- or quasibinomial regressions; Section 1.3–1.5 in S1 File), also perform much worse. Therefore, the results reported here are based on standard Gaussian regression. In turn, while using Eq (2) is in line with the principles of how the data ought to be analyzed and Eq (2) is not, it turns out that the random effects associated with the repeated measurements on individual dogs are weak, and the qualitative outcome is the same both in the mixed model of Eq (2) and the straightforward two-way ANOVA of Eq (1). That said, here we will report results from the mixed model.

Finally, to make sure that the results obtained via fitting Eqs (1) and (2) are robust to changing model assumptions, we also used a non-parametric model (the Scheirer-Ray-Hare test [19]) for interpreting the data. This procedure is related to two-way ANOVA in the same way as the Kruskal-Wallis test is related to one-way ANOVA: it fulfils the same purpose but in a non-parametric setting. The results from the Scheirer-Ray-Hare test turn out to be fully in line with those from the regression models described above.

### Results

**Fig 2:**
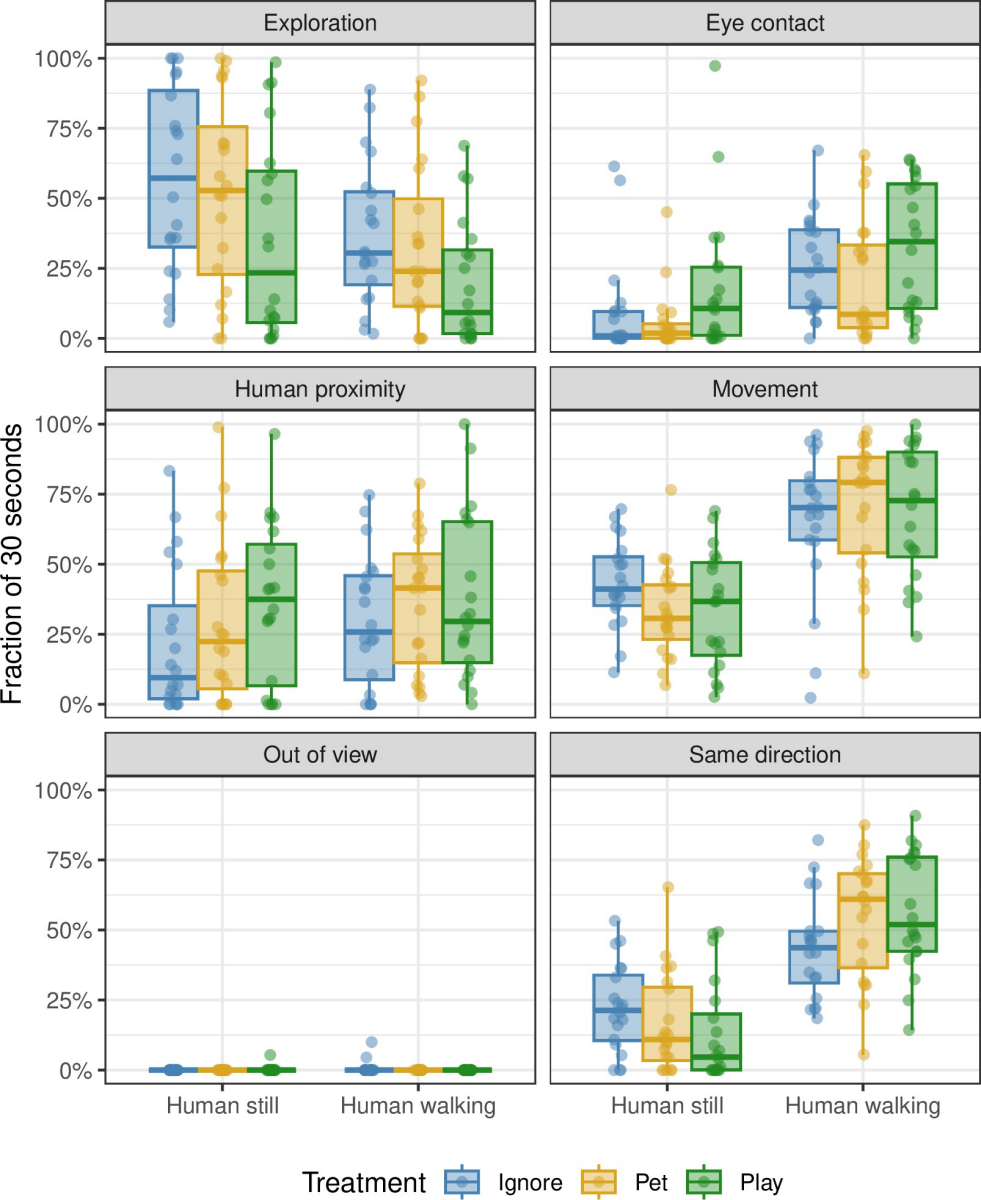
The effect of previous human interaction and of walking/standing phase on dog behaviour during the behavioural synchronisation test. The fraction of time out of the total 30 seconds (y-axis) spent by dogs on each of five activities (exploration, eye contact, human proximity, movement, and same direction; see panel labels). These fractions were measured for all three treatments of ignore (blue), pet (yellow) and play (green) and whether the owner was standing still or walking (x-axis). Each point corresponds to one dog’s measurement; the box plots summarize these points.

During the walking phase, the dogs were moving more (p = 0.0003), were more in the same direction as the owner (p = 0.0001) and showed more eye-contact behaviour (p = 0.0012) compared to the standing still phase (Fig 2; S1 File). By contrast, they displayed less exploration during the walking phase compared to the standing still phase (p = 0.0028; Fig 2). Table 2 summarizes all results in more detail, with further information in S1 File.

**Table 2:** Summary of results from the linear mixed models of Eq (2). For each of the behaviours (first column), we have the model coefficients in the second column (but without the intercept), along with their estimate (3rd column), standard error (4th column), and the associated p-value (5th column). Bold font highlights significant results.

Behaviour	Term	Estimate	Standard error	p-value
Human proximity	phaseWalking	0.079	0.066	0.2313
	trtPet	0.072	0.066	0.2789
	trtPlay	0.142	0.066	**0.0319**
	phaseWalking:trtPet	-0.007	0.093	0.9415
	phaseWalking:trtPlay	-0.064	0.093	0.4952
Same direction	phaseWalking	0.212	0.055	**0.0001**
	trtPet	-0.056	0.055	0.3112
	trtPlay	-0.092	0.055	0.0939
	phaseWalking:trtPet	0.168	0.078	**0.0301**
	phaseWalking:trtPlay	0.225	0.078	**0.0038**
Exploration	phaseWalking	-0.197	0.066	**0.0028**
	trtPet	-0.050	0.066	0.4446
	trtPlay	-0.216	0.066	**0.0010**
	phaseWalking:trtPet	0.005	0.093	0.9584
	phaseWalking:trtPlay	0.042	0.093	0.6553
Eye contact	phaseWalking	0.159	0.049	**0.0012**
	trtPet	-0.038	0.049	0.4409
	trtPlay	0.085	0.049	0.0821
	phaseWalking:trtPet	-0.005	0.069	0.9386
	phaseWalking:trtPlay	-0.013	0.069	0.8549
Movement	phaseWalking	0.221	0.061	**0.0003**
	trtPet	-0.104	0.061	0.0902
	trtPlay	-0.096	0.061	0.1171
	phaseWalking:trtPet	0.157	0.087	0.0696
	phaseWalking:trtPlay	0.129	0.087	0.1380

The type of human interaction treatment (*Ignore/Pet/Play*), and especially *Play* treatment (p = 0.009), but also *Pet* treatment (p = 0.048), positively affected effect the direction of the dog during the walking phase (Fig 2; S1 File). Hence, the dogs were more in the same direction as their owners after these treatments. *Play* treatment also decreased the overall exploration behaviour (p = 0.026). Proximity to owner was not affected by either treatment or phase (p > 0.05). Also, the age of the dog did not explain any behavioural variations (p > 0.1; S1 File). See Table 3 for total durations of synchronisation-related behaviours.

**Table 3:** Total duration (% of 60 s) of the synchronisation-related behaviours in the different treatments.

Behaviour	Treatment	Duration (% ± SE)
Human proximity	Ignore	26.0 ± 3.9
	Pet	32.8 ± 4.2
	Play	36.9 ± 4.5
Eye contact	Ignore	17.5 ± 3.0
	Pet	13.4 ± 2.9
	Play	25.4 ± 4.0
Same direction as owner	Ignore	32.8 ± 3.1
	Pet	35.6 ± 4.4
	Play	34.8 ± 4.6

## Study B—different breed groups

### Methods

#### Ethics declarations

In this study, we exclusively investigated the behaviour of privately owned dogs. No special ethical permission for use of privately owned dogs in non-invasive observational studies is required in Sweden (SJVFS 2019:9, 2 ch., 17§), nevertheless dog owners gave their written consent to voluntarily participate in the study which was executed in Linköping, Sweden. All methods were performed in accordance with the relevant guidelines and regulations and no animal displayed any signs of stress during the behavioural synchronisation. The sample size and animal information have been reported in accordance with ARRIVE guidelines (www.arriveguidelines.org). Additionally, consent to publish the photo in Fig 1 was obtained from the dog owner.

#### Subjects

Twenty-four dogs, consisting of ancient dog breeds (15 females and 9 males) such as Shiba Inu, Basenji, and Siberian Husky, and 17 solitary hunting dogs (14 females and 3 males) such as Swedish and Norwegian Elkhound and Dachshund, were recruited for the study through social media and personal contacts. The age range of the dogs was from 1–15 years. Additionally, the companion dogs from various breeds that were part of from Study A were included, but only the test which did not include any human interaction (ignore treatment). All dogs lived indoors as pet dogs and regularly walked, although the solitary hunting dogs were also actively used for hunting purposes. A breed was considered to be of an ancient dog breed if it was thought to be genetically closer to wolves [20]. More detailed information about the dogs and their specific breeds can be found in S1 Table. The experiments were conducted outdoors at Linköping University in the southeast of Sweden during September and October in 2019.

#### Experimental procedure

The experimental procedure in Study B was identical to Study A, with the exception that the dogs in Study B only performed the test procedure once without any specific treatment or interaction, equivalent to the ignore treatment in Study A. This modification was made because the focus in Study B was to investigate possible breed differences rather than the effect of human interactions. After the initial walk on loose leash around the test arena, the dyads walked out of the arena, and the owner received instructions about the test procedure from the test leader while ignoring the dog. Then, similar to Study A, they walked into the starting area, the leash was removed from the dog and given to the test leader, and the owner was provided with a timer and a map. If the owner felt uncomfortable having the dog off-leash within the test arena, they were given the option to use a 10-meter-long leash, which was loose on the ground (used by 3 ancient dog breed owners and 4 solitary hunting dog owners). The test started when the owner reached the first designated standing still position (Fig 1). All dyads were video recorded during the test for later analysis using Observer XT (Noldus) software, using a predetermined ethogram (Table 1).

#### Data analysis

The methods of analysis are identical to those in Study A, with one exception: since here the animals do not go through repeated measures, there is no need to use mixed-effect models. We therefore use just the fixed-effect linear model (two-way ANOVA) and the Scheirer-Ray-Hare test, with the predictors being phase (human still or human walking) and breed type (companion, hunting, or ancient dog breeds). The model reads as follows:

$${\left(\mathrm{fraction}\right)}_{i}=\beta_{0}+\beta_{1}{\left(\mathrm{phase}\right)}_{i}+\beta_{2}{\left(\mathrm{group}\right)}_{i}+\beta_{3}{\left(\mathrm{phase}\right)}_{i}{\left(\mathrm{group}\right)}_{i}+\epsilon_{i},$$ (3)

where “group” refers to breed type. The detailed analysis is in Section 2 in S1 File. Results across the linear model and the non-parametric Scheirer-Ray-Hare test are consistent with one another; the results reported below are based on the former.

### Results

**Fig 3:**
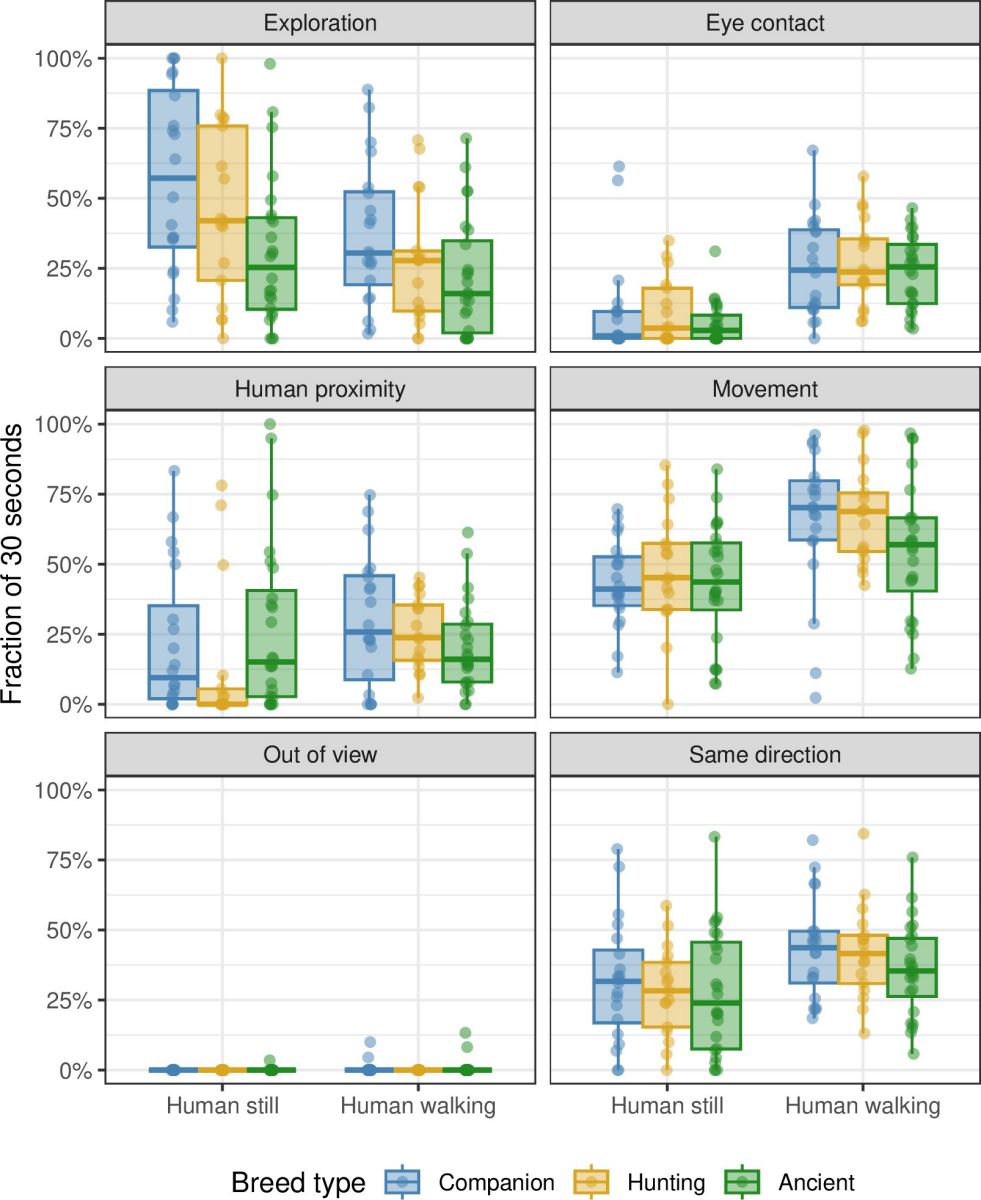
The effect of different breed groups and of walking/standing phase on dog behaviour during the behavioural synchronisation test. The fraction of time out of the total 30 seconds (y-axis) spent by dogs on each of five activities (exploration, eye contact, human proximity, movement, and same direction; see panel labels). The dogs were either companion dogs (blue), hunting dogs (yellow), or from an ancient breed (green). The owner was either standing still or walking (x-axis). Each point corresponds to one dog’s measurement; the box plots summarize these points.

Dogs, regardless of the group they belonged to, spent more time moving (p = 0.002), looked longer durations at their owner (p = 0.016), and tended to be more in the same direction as their owner (p = 0.058) when their owner was walking, as opposed to when the owner was standing still (Fig 3; S1 File). On the other hand, the dogs explored less during the walking phase compared to the standing still phase (p = 0.022). Among the different groups, dogs belonging to the ancient breeds explored the least (Fig 3; S1 File). See Table 4 for detailed statistics and Table 5 for total durations of synchronisation-related behaviours.

**Table 4:** Summary of results from the linear models of Eq (3). For each of the behaviours (first column), we have the model coefficients in the second column (but without the intercept), along with their estimate (3rd column), standard error (4th column), and the associated p-value (5th column). Bold font highlights significant results.

Behaviour	Term	Estimate	Standard error	p-value
Human proximity	phaseWalking	0.079	0.074	0.2893
	groupHunting	-0.090	0.078	0.2453
	groupAncient	0.052	0.071	0.4669
	phaseWalking:groupHunting	0.048	0.110	0.6601
	phaseWalking:groupAncient	-0.146	0.100	0.1480
Same direction	phaseWalking	0.115	0.060	0.0581
	groupHunting	-0.037	0.063	0.5543
	groupAncient	-0.040	0.058	0.4873
	phaseWalking:groupHunting	0.025	0.089	0.7774
	phaseWalking:groupAncient	-0.033	0.081	0.6884
Exploration	phaseWalking	-0.197	0.085	**0.0219**
	groupHunting	-0.117	0.088	0.1867
	groupAncient	-0.262	0.081	**0.0016**
	phaseWalking:groupHunting	0.013	0.125	0.9150
	phaseWalking:groupAncient	0.107	0.115	0.3512
Eye contact	phaseWalking	0.159	0.045	**0.0006**
	groupHunting	-0.002	0.047	0.9609
	groupAncient	-0.042	0.043	0.3323
	phaseWalking:groupHunting	0.018	0.066	0.7815
	phaseWalking:groupAncient	0.025	0.061	0.6747
Movement	phaseWalking	0.220	0.068	**0.0015**
	groupHunting	0.045	0.071	0.5231
	groupAncient	-0.004	0.065	0.9532
	phaseWalking:groupHunting	-0.030	0.100	0.7648
	phaseWalking:groupAncient	-0.100	0.092	0.2764

**Table 5:** Total duration (% of 60 s) of the synchronisation-related behaviours in the different breed groups.

Behaviour	Breed group	Duration (% ± SE)
Human proximity	Companion breed group	26.0 ± 3.9
	Ancient dog breeds	23.8 ± 3.5
	Solitary hunting breeds	19.3 ± 3.7
Eye contact	Companion breed group	17.7 ± 3.0
	Ancient dog breeds	17.5 ± 3.8
	Solitary hunting breeds	18.1 ± 2.8
Same direction as owner	Companion breed group	37.6 ± 3.3
	Ancient dog breeds	32.0 ± 2.9
	Solitary hunting breeds	35.2 ± 3.0

## Study C—similarly socialised pack-living wolves and dogs

### Methods

#### Ethics declarations

The behavioural experiment conducted at the Wolf Science Centre (WSC; www.wolfscience.at/en) was carefully planned in collaboration with the WSC, and all experimental protocols were approved by the Ethics and Animal Welfare Committee of the University of Veterinary Medicine, Vienna, in accordance with the University’s guidelines for Good Scientific Practice (Protocol number: ETK-154/10/2021). All methods were performed in accordance with the relevant guidelines and regulations. Informed consent was obtained from the female handlers who participated in the study. None of the included animal showed signs of stress during the behavioural synchronisation test, and sample size and animal information have been reported in accordance with ARRIVE guidelines (www.arriveguidelines.org).

#### Subjects

Six wolves and six mixed breed dogs, all living in their respective packs, were housed and tested at the WSC in Ernstbrunn, Austria, in November 2021. The animals’ ages varied between 7 and 12 years, and they were all born in captivity and hand raised and socialised in a comparable manner by experienced animal professionals. The two female handlers who acted as owners in this experiment had been extensively working with the animals, maintaining relationships that spanned between 7–12 years. The wolves and dogs were living in pairs or groups within spacious enclosures, and they were used to being walked on leash e.g. between enclosures. Additional information about the individual wolves and dogs can be found in S1 Table.

#### Experimental procedure

All animals were individually tested, and since the wolves and dogs did not have owners, they were tested with one of two female handlers who had the longest relationships with the animals. In order to test the animals separately, each animal was walked (on a leash) by the handler to another enclosure for the testing. The animal was allowed to acclimatise a couple of minutes before the test so when the handler entered the enclosure the tested animal was already present. The handler walked directly to the start position, stood still, and followed a predetermined walking and standing scheme similar to that used in study A and B (Fig 1A). To avoid distracting the animals with novel fences, the test procedure took place in one of the corners of a large enclosure. Also, no extra signs were placed on the ground for the same reason. Therefore, two sides of the test arena were without a fence, allowing the animals to move away from the experimental arena.

When the handler entered the enclosure, the sole animal present approached her. The handler was instructed to ignore the animal, walk to the first standing still position, and follow the designated walking and standing still scheme. Since the final part of the scheme was intended to be near to the outer edges of the test arena (as opposed to in the middle of the arena during the first walking phase), the handler turned towards the fence during the second walking phase (blue line in Fig 1A). Consequently, the last turn in Fig 1A could be in a clockwise direction. All dyads were video recorded for later analysis in Observer XT (Noldus) software using a predetermined ethogram (Table 1). However, due to the greater distance and more fences between the camera and the test arena, the behaviours eye contact and exploration were challenging to assess from the videos. Therefore, these behaviours were not analysed any further.

#### Data analysis

The methods of analysis are identical to those in Study B, except the predictors are phase (human still or human walking) and species (dog or wolf). The detailed analysis is in Section 3 in S1 File. Diagnostic plots reveal that the assumptions behind the linear regression are not satisfied very well. As such, one can have more confidence in the results of the non-parametric Scheirer-Ray-Hare test—but those are in full agreement with those of the linear model.

### Results

**Fig 4:**
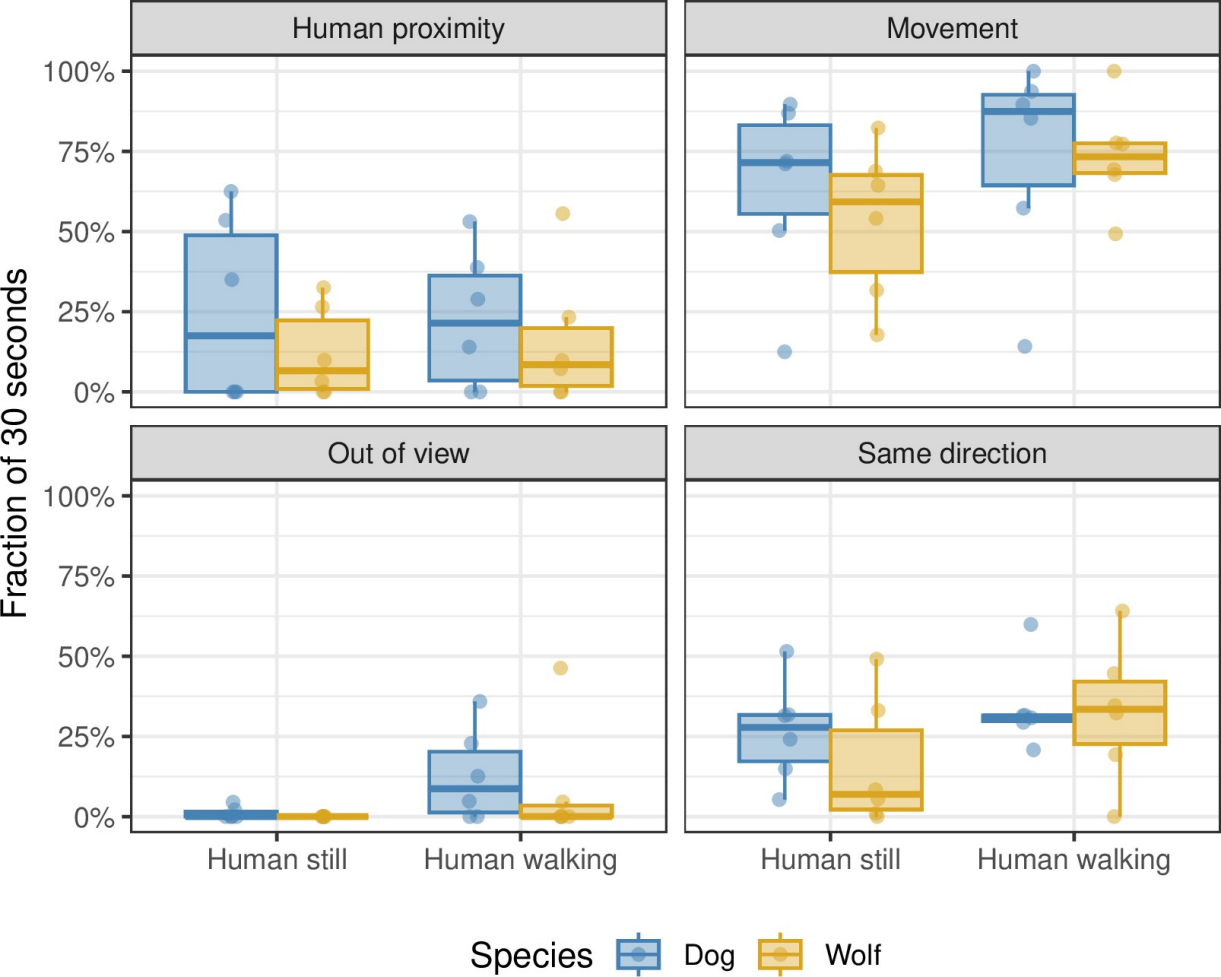
The effect of domestication and of walking/standing phase on dog and wolf behaviour during the behavioural synchronization test. The fraction of time out of the total 30 seconds (y-axis) spent by dogs (blue) and wolves (yellow) on each of three activities (human proximity, movement, and same direction; see panel labels). A human was either standing still or walking (x-axis). Each point corresponds to one measurement; the box plots summarize these points.

The wolves and socialised dogs did not change their moving behaviour, their direction in relation to the handler, or human proximity durations between the standing still and walking phases of the handler (p > 0.1 from both the linear model and the Scheirer-Ray-Hare test; Fig 4; S1 File). See Table 6 for total durations of synchronisation-related behaviours.

**Table 6:** Total duration (% of 60 s) of the synchronisation-related behaviours in socialised dogs and wolves.

Behaviour	Species	Duration (% ± SE)
Human proximity	Socialised dogs	23.8 ±7.0
	Socialised wolves	14.0 ± 5.0
Same direction as owner	Socialised dogs	30.2 ± 4.2
	Socialised wolves	24.3 ± 6.3

### Discussion

The objective of our study was to investigate the interspecies behavioural synchronisation of different groups of dogs and wolves together with their owners/handlers. Additionally, we aimed to compare the synchronisation-related behaviours following various human interactions. Our results showed that all dogs that live indoors as pets, exhibited behavioural synchronisation with their owner. They also increased their eye contact-seeking behaviour when the owner start walking. Moreover, we found that even brief interactions of play and petting increased the dogs’ probability to be in the same direction as their owner. However, in contrast to indoor living pet dogs, socialised pack-living dogs and wolves did not demonstrate synchronisation in activity.

The main characteristic of behavioural synchronisation is the adjustment of activity pace in response to the presence of others [9,10]. In our study A and B, we found that dogs moved more when their owners were moving compared to when their owners were standing still, regardless of the human-animal interaction prior to the test or breed group. These findings align with previous studies that reported dogs [12] including shelter dogs [13], synchronising their activity with their owner/handler. Cimarelli et al. [15] also observed movement synchronisation within both pack-living wolves and pack-living dogs, although dogs displayed stronger synchronised with each other. Notably, the quality of the relationship between individuals was significantly associated with the degree of synchronisation in both species. In our study C, we did not find movement synchronisation with the handler for either the pack-living dogs or the wolves. The differences in moving synchronisation between companion dogs and pack living wolves and dogs may be linked to variations in the quality of the human-animal relationship and should be explored further in future studies.

Proximity to the owner/handler can indeed be closely related to behavioural synchronisation [9,10]. However, in our study, we found that the pace of the human did not influence the proximity of dogs or wolves to their owner/handler. The duration of proximity to the owner/handler remained similar between the walking and standing still phases, regardless of human interaction treatment (study A), breed group (study B), or species (study C). Interestingly, in study C, the socialised pack-living dogs showed similar duration of proximity to humans as the indoor-living pet dogs in study B. This suggests that even though the pack-living dogs were able to leave the test arena and explore the rest of the enclosure, they stayed in human proximity. However, it is important to note that this comparison was not statistically tested due to the differences in experimental settings, and any conclusions should be drawn cautiously. This would also contrast with Duranton et al. [13], where shelter dogs exhibited less proximity to humans compared to pet dogs. Further research is needed to explore these differences among different groups of dogs more extensively.

Being in the same direction could also contribute to behavioural synchronisation [14]. Interestingly, we found that the total duration spent in the same direction as the human was similar in the pack-living dogs in study C and the indoor-living dogs of ancient dog breeds in study B. This might suggest a small degree of synchronisation even in pack-living socialised dogs. Furthermore, both in study A and B, dogs were more in the same direction as their owner and showed longer duration of eye contact when the owner was walking compared to when the owner was standing still. Apart from a synchronisation effect, it could be speculated that this behaviour change is influenced by the dog’s previous life experiences. The beginning of each walking phase by the owner might resemble a typical dog walk, triggering the dog’s expectation and resulting in increased synchronisation behaviours. However, such speculations cannot be resolved within the scope of this study.

In our study A, we found that human interactions with the dog prior to the test could have an impact on the dog’s behaviour. Specifically, *Play* and *Pet* interactions led to an increase of the dogs’ probability to be in the same direction as their owner, while exploration decreased after the *Play* treatment. Previous studies have shown that play sessions can enhance the dog’s attention towards their play partner [21]. The dogs in study A and B in our study did increase their eye-contact seeking behaviour when the owner start walking but contrary to our hypothesis, the duration was not significantly increased after *Play* interaction in study A. A visual inspection of Fig 2 might suggest that play could have some effect at least on some of the dogs but that has to be investigated further, and maybe divided by breed groups. Still, our findings from study A suggest that even small efforts, such as engaging in play or pet interactions, can alter the dog’s behaviour, and in our case, the probability to align with humans.

The duration of exploration behaviour was influenced not only by treatment in study A but also by the breed group in study B, yielding unexpected results. Surprisingly, it was the companion dogs that exhibited the longest exploration durations. However, there are some confounding factors that should be considered when interpreting this result. Firstly, the dogs in the companion group were tested during the spring, while the hunting dogs and the dogs of ancient breed groups were tested in late summer/autumn. Therefore, the seasonal variation may have influenced the dogs’ exploratory behaviour, but this is only speculative. Secondly, a common confounding factor in canine studies is the presence of odors from previous participants, which could potentially distract the following participants. This was also a concern in study C, where the animals could leave the unfenced sides of the test arena and explore tracks of other individuals. Due to these factors, we did not do statistical comparisons between study A, B and C. However, future studies should aim to investigate these different groups in identical environments to provide more conclusive findings.

In conclusion, our findings align with previous research, demonstrating that indoor-living dogs synchronise their behaviour with their owners, whereas pack-living socialised dogs and wolves did not display synchronisation in activity. Interestingly, the pack-living dogs showed comparable durations of human proximity to the pet dogs, highlighting the need for further comparative studies between these groups. Additionally, our study revealed that even very little play and pet interactions was enough to increase the dogs’ alignment with its owner.

## Supporting information

S1 Table Supplementary animal information. (XLSX)

S2 Table Behavioural data from Study A. (CSV)

S3 Table Behavioural data from Study B. (CSV)

S4 Table Behavioural data from Study C. (CSV)

S1 File Supplementary statistical analysis. Explanations including R codes, test statistics and figures.
(PDF)

S2 File Metadata for S2–S4 Tables. (TXT)
