# Supplementary material for: Behavioural synchronisation between different groups of dogs and wolves and their owners/handlers: Exploring the effect of breed and human interaction
Source: PLoS One. 2024 May 3;19(5):e0302833. doi: 10.1371/journal.pone.0302833 (PMC11068182; doi:10.1371/journal.pone.0302833)
Supplement: S1 File — Explanations including R codes, test statistics and figures. (PDF) [file pone.0302833.s005.pdf]

# Behavioural synchronisation between different groups of dogs and wolves and their owners/handlers: exploring the effect of breed and human interaction

Supplementary statistical analysis

Jasmine Heurlin, György Barabás & Lina S. V. Roth

All analyses were done using R version 4.2.2[1] (see full session information in Section 4). For reproducibility, below we always show the R code performing the discussed analyses, along with the output generated by that code.

## 1 Study A

### 1.1 Loading and tidying the data

We first load the data. We drop unnecessary columns, and organize all columns that measure the duration of various behaviours exhibited by the dogs into key-value pairs:

```
# Load two packages:
library(tidyverse) # Efficient data manipulation and plotting
library(ggfortify) # Diagnostic plots for linear models

studyA <- read_csv("study-A.csv") |>
  # Drop unnecessary columns:
  select(!ends_with("sex")) |>
  # Arrange measured behavioural variables in key-value pairs:
  pivot_longer(cols = humanprox:outview,
               names_to = "behaviour",
               values_to = "fraction") |>
  # Capitalized entries in `phase` and `treatment`:
  mutate(across(c(phase, treatment), str_to_title)) |>
  # Human-readable names for the various dog behaviours:
  mutate(behaviour = case_match(
    behaviour,
    "humanprox" ~ "Human proximity",
    "direction" ~ "Same direction",
    "exploration" ~ "Exploration",
    "eyecon" ~ "Eye contact",
    "move" ~ "Movement",
```

[1] R Core Team (2022). R: A language and environment for statistical computing. R Foundation for Statistical Computing, Vienna, Austria. URL https://www.R-project.org/.

```
    "outview" ~ "Out of view",
    .default = behaviour
  ))
```

Here are the first few rows of this cleaned table:

```
studyA |>
  head(n = 12) |>
  knitr::kable()
```

| id | phase | treatment | order | dogage | behaviour | fraction |
|---|---|---|---|---|---|---|
| 1 | Still | Ignore | 3 | 3 | Human proximity | 0.543 |
| 1 | Still | Ignore | 3 | 3 | Same direction | 0.109 |
| 1 | Still | Ignore | 3 | 3 | Exploration | 0.240 |
| 1 | Still | Ignore | 3 | 3 | Eye contact | 0.207 |
| 1 | Still | Ignore | 3 | 3 | Movement | 0.283 |
| 1 | Still | Ignore | 3 | 3 | Out of view | 0.000 |
| 1 | Still | Pet | 1 | 3 | Human proximity | 0.672 |
| 1 | Still | Pet | 1 | 3 | Same direction | 0.050 |
| 1 | Still | Pet | 1 | 3 | Exploration | 0.430 |
| 1 | Still | Pet | 1 | 3 | Eye contact | 0.236 |
| 1 | Still | Pet | 1 | 3 | Movement | 0.283 |
| 1 | Still | Pet | 1 | 3 | Out of view | 0.000 |

We can now visualize these data (Figure 1):

```
# Function to aid plotting raw data:
plotRawData <- function(data, column, label) {
  data |>
    # Rename labels, to show better on the plot:
    mutate(phase = ifelse(phase == "Walking", "Human walking",
                          "Human still")) |>
    # Create plot:
    ggplot(aes(x = phase, y = fraction,
               colour = {{column}}, fill = {{column}})) +
    geom_boxplot(position = "dodge", alpha = 0.4, outlier.shape = NA) +
    geom_point(alpha = 0.5,
               position = position_jitterdodge(jitter.width=0.05, seed=63)) +
    facet_wrap(~ behaviour, ncol = 2) +
    scale_x_discrete(name = "Action of human") +
    scale_y_continuous(name = "Fraction of 30 seconds",
                       labels = scales::percent) +
    scale_colour_manual(name = label,
                        values = c("steelblue","goldenrod","forestgreen")) +
    scale_fill_manual(name = label,
                      values = c("steelblue","goldenrod","forestgreen")) +
    theme_bw() +
    theme(axis.title.x = element_blank(), legend.position = "bottom")
```

```
}

# Plot data for all behaviours:
studyA |>
  plotRawData(column = treatment, label = "Treatment")
```

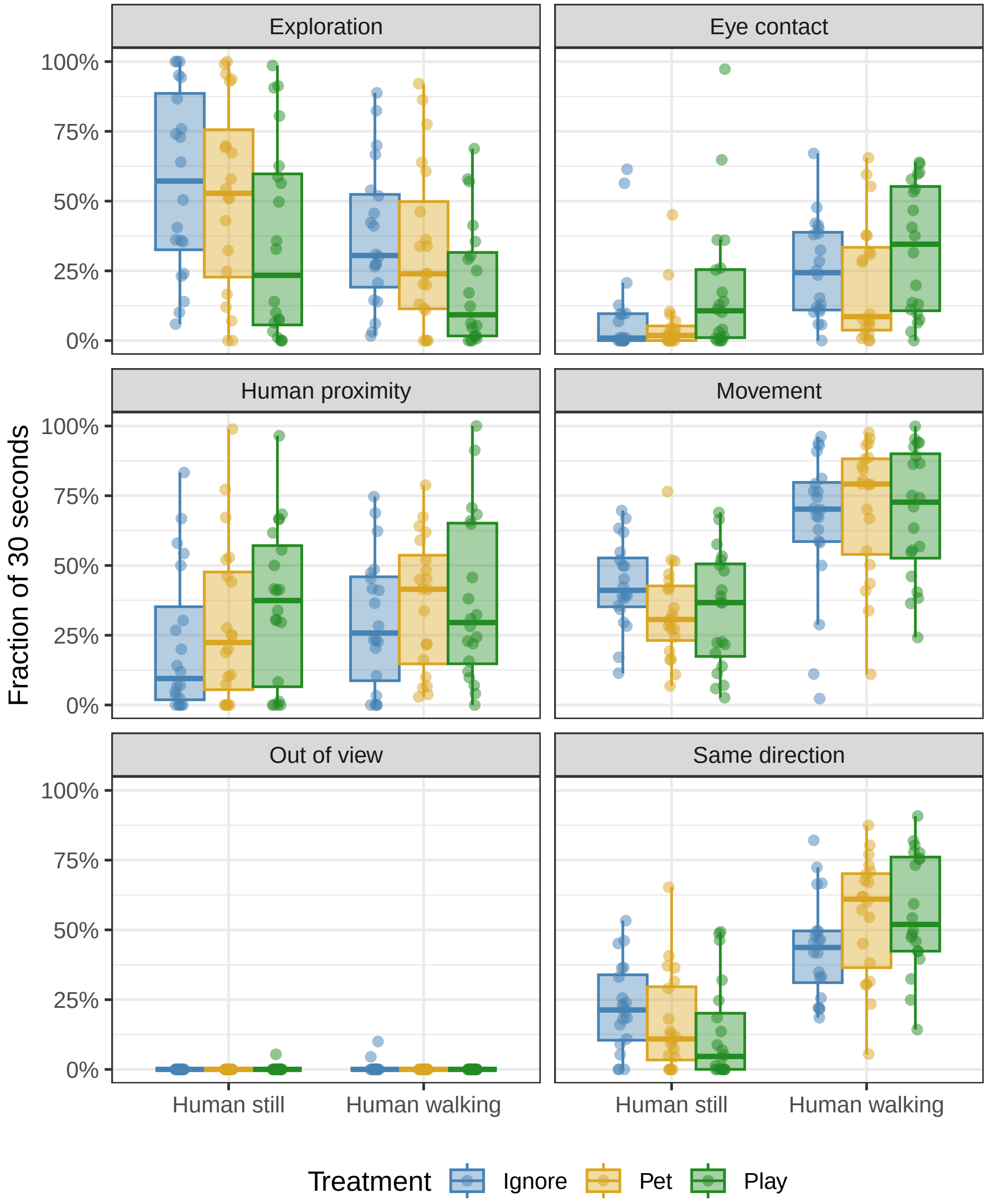


Figure 1: The fraction of time out of the total 30 seconds (y-axis) spent by dogs on each of six behaviours (panel labels). These fractions were measured for all three treatments of ignore/pet/play (colours) and whether the owner was standing still or walking (x-axis). Each point corresponds to the measurement of one dog; the box plots summarize these points.

As seen, the data for being out-of-view are pathological, with almost all measurements being zero. For this reason, it will be excluded from our subsequent analyses.

### 1.2 Dog age as a predictor

One question is whether the dogs' ages are important in predicting any of the patterns. The following analysis suggests that they aren't. Plotting all measured durations against dog age, we get Figure 2. Given the p-values of the regression slopes (shown in the figure), dog age does not appear to play a significant role in explaining variation in the behaviours.

```r
studyA |>
  # Create a sub-table of the data for each behaviour:
  nest(data = !behaviour) |>
  # Simple linear regression of fraction on age, in each:
  mutate(fit = map(data, \(x) lm(fraction ~ dogage, data = x)),
         fit = map(fit, broom::tidy)) |>
  unnest(fit) |>
  # Drop the intercept (to help print p-values in plot):
  filter(term != "(Intercept)") |>
  # Keep only the relevant columns:
  select(behaviour, data, p.value) |>
  unnest(data) |>
  # Extract p-values (rounded to two digits) in separate column:
  mutate(p.value = str_c("p = ", round(p.value, 2))) |>
  # Create plot:
  ggplot(aes(x = dogage, y = fraction)) +
  geom_point(colour = "steelblue", alpha = 0.2) +
  geom_smooth(method = lm, se = FALSE,
              colour = "goldenrod", linewidth = 0.5) +
  geom_text(data = . %>% select(behaviour, p.value) %>% distinct(),
            aes(label = p.value), x = 2, y = 0.9) +
  scale_x_continuous(name = "Age of dog (years)") +
  scale_y_continuous(name = "Fraction of 30 seconds",
                     labels = scales::percent, limits = c(0, 1)) +
  facet_wrap(~behaviour, ncol = 2) +
  theme_bw()
```

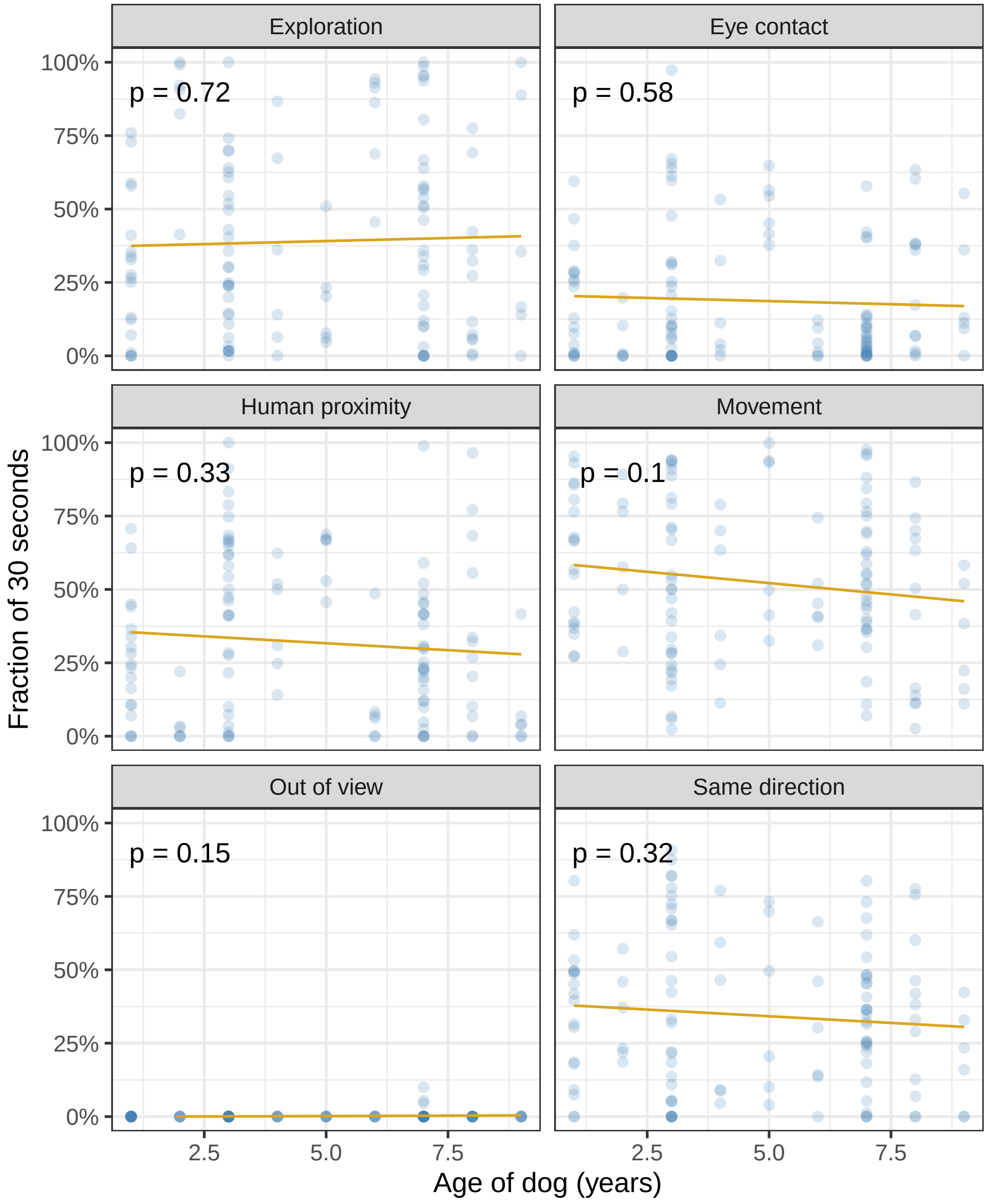


Figure 2: Simple linear regression of the fraction of time (y-axis) on age (x-axis), separately for each behaviour (panels).

### 1.3 Fitting generalized linear models

The fraction of time spent with a given behaviour is a value between 0 and 1. So it would be natural to try to perform regression using a generalized linear model with a family which restricts the response variable to the 0-to-1 range, such as quasibinomial regression. Additionally, it would be good to take the zero-inflation[2] of the data into account as well. It turns out however that in our case, these are not necessary, and Gaussian regression works best. We will see below in Section 1.5 that both binomial and Gamma regressions perform worse than Gaussian regression,

[2]There is also one-inflation in the data, but only for five data points across the entire dataset.

and that accounting for zero-inflation does not improve the model fit (this latter point is likely due to zero-inflation not being too prevalent in the data, as seen in Figure 1). Furthermore, diagnostic plots reveal that the assumptions of the Gaussian model are reasonably well satisfied.

We therefore fit Gaussian linear models for each behaviour. The fixed-effect predictors are treatment, phase, and their interaction. We fit two versions of the model: the first only includes these fixed-effect predictors; the second includes random effects as well. The random effects account for each dog undergoing repeated measures: they participate in all three treatments and the two phases (human still or walking) of the experiment. This can be modeled by adding a random intercept that depends on the identity of the dog. We fit these models to the data:

```
regrDatA <- studyA |>
  # Remove the undesired out-of-view behaviour:
  filter(behaviour != "Out of view") |>
  # Shorten the column name `treatment` to `trt` for convenience:
  rename(trt = treatment) |>
  # Create a sub-table of the data for each behaviour:
  nest(data = !behaviour) |>
  # Fit linear model with fixed effects for each behaviour:
  mutate(fixedEffects = map(data, \(x) {
    lm(fraction ~ phase * trt, data = x)
  } )) |>
  # Do the same, but with a random intercept for each dog:
  mutate(randomEffects = map(data, \(x) {
    lme4::lmer(fraction ~ phase * trt + (1|id), data = x)
  } ))
```

Obtaining parameter-specific p-values for the random-effect models is not straightforward, due to the fact that the degrees of freedom are not easily computed. One possible workaround is to use a normal approximation, assuming that the degrees of freedom is large enough for the $t$-distribution to have converged to the $z$-distribution with sufficient accuracy. While this method will produce p-values that are overly optimistic, this is not a huge problem as long as the sample sizes aren't very small.[3] As we will see, the p-values obtained in this way are qualitatively the same as for the fixed-effect models.

With that, we generate the regression tables. Each `term` (parameter) has its `estimate` and its standard error listed, under `std. error (fixed)` and `std. error (random)`, referring to the fixed- and random-effect models. Finally, `p-value (fixed)` and `p-value (random)` show the same for the computed p-values:

```
regrDatA |>
  # Standardize table and column names for fixed-effect models:
  mutate(ftab = map(fixedEffects, \(x) broom::tidy(x) |>
                      select(!statistic) |>
                      mutate(type = "fixed") |>
                      rename(stdError = std.error, pvalue = p.value)
  )) |>
  # Standardize table and column names for random-effect models:
```

[3] Barr, D. J., Levy, R., Scheepers, C., and Tily, H. J. (2013). Random effects structure for confirmatory hypothesis testing: Keep it maximal. *J. Mem. Lang.* 68, 255–278. doi: 10.1016/j.jml.2012.11.001

```
mutate(rtab = map(randomEffects,
                  compose(as_tibble,data.frame,coef,summary))) |>
mutate(rtab = map2(ftab, rtab,
                   \(f, r) mutate(r, term = f$term, .before = 1) |>
                     # Estimate p-values via normal approximation:
                     mutate(p.value = 2*(1-pnorm(abs(t.value)))) |>
                     mutate(type = "random") |>
                     select(!t.value) |>
                     rename(estimate = Estimate,
                            stdError = Std..Error,
                            pvalue = p.value)
)) |>
# For each behaviour, join the fixed- and random-effect tables:
mutate(jtab = map2(ftab, rtab, \(f, r) {
  bind_rows(f, r) |>
    # Arrange in human-readable form:
    pivot_wider(names_from = type,
                values_from = c(estimate, stdError, pvalue)) |>
    # `estimate_random` same as `estimate_fixed`:
    select(!estimate_random) |>
    # Human-readable column names:
    rename(estimate = estimate_fixed,
           `std. error (fixed)` = stdError_fixed,
           `std. error (random)` = stdError_random,
           `p-value (fixed)` = pvalue_fixed,
           `p-value (random)` = pvalue_random) |>
    mutate(term = ifelse(term == "(Intercept)", "intercept", term)) |>
    mutate(across(!starts_with("p-") & !term, \(x) round(x, 3))) |>
    mutate(across(starts_with("p-"), \(x) round(x, 4)))
} )) |>
# Loop through each joint table and display them:
mutate(walk2(behaviour, jtab, \(m, tab) {
  cat(str_c(m, ":")) # Behaviour name
  knitr::kable(tab) |> # The corresponding table
    #kableExtra::kable_styling(font_size = 10) |>
    print()
} )) |>
# Avoid using the resulting table as the output:
invisible()
```

Human proximity:

| term | estimate | std. error (fixed) | std. error (random) | p-value (fixed) | p-value (random) |
|---|---|---|---|---|---|
| intercept | 0.220 | 0.060 | 0.060 | 0.0004 | 0.0002 |
| phaseWalking | 0.079 | 0.085 | 0.066 | 0.3538 | 0.2313 |
| trtPet | 0.071 | 0.085 | 0.066 | 0.4014 | 0.2789 |

| term | estimate | std. error (fixed) | std. error (random) | p-value (fixed) | p-value (random) |
|---|---|---|---|---|---|
| trtPlay | 0.142 | 0.085 | 0.066 | 0.0978 | 0.0319 |
| phaseWalking:trtPet | -0.007 | 0.120 | 0.093 | 0.9546 | 0.9415 |
| phaseWalking:trtPlay | -0.064 | 0.120 | 0.093 | 0.5968 | 0.4952 |

Same direction:

| term | estimate | std. error (fixed) | std. error (random) | p-value (fixed) | p-value (random) |
|---|---|---|---|---|---|
| intercept | 0.222 | 0.042 | 0.042 | 0.0000 | 0.0000 |
| phaseWalking | 0.212 | 0.060 | 0.055 | 0.0005 | 0.0001 |
| trtPet | -0.056 | 0.060 | 0.055 | 0.3525 | 0.3112 |
| trtPlay | -0.092 | 0.060 | 0.055 | 0.1253 | 0.0939 |
| phaseWalking:trtPet | 0.168 | 0.084 | 0.078 | 0.0480 | 0.0301 |
| phaseWalking:trtPlay | 0.225 | 0.084 | 0.078 | 0.0087 | 0.0038 |

Exploration:

| term | estimate | std. error (fixed) | std. error (random) | p-value (fixed) | p-value (random) |
|---|---|---|---|---|---|
| intercept | 0.569 | 0.068 | 0.068 | 0.0000 | 0.0000 |
| phaseWalking | -0.197 | 0.096 | 0.066 | 0.0425 | 0.0028 |
| trtPet | -0.050 | 0.096 | 0.066 | 0.6012 | 0.4446 |
| trtPlay | -0.216 | 0.096 | 0.066 | 0.0263 | 0.0010 |
| phaseWalking:trtPet | 0.005 | 0.136 | 0.093 | 0.9715 | 0.9584 |
| phaseWalking:trtPlay | 0.041 | 0.136 | 0.093 | 0.7601 | 0.6553 |

Eye contact:

| term | estimate | std. error (fixed) | std. error (random) | p-value (fixed) | p-value (random) |
|---|---|---|---|---|---|
| intercept | 0.095 | 0.045 | 0.045 | 0.0346 | 0.0324 |
| phaseWalking | 0.159 | 0.063 | 0.049 | 0.0133 | 0.0012 |
| trtPet | -0.038 | 0.063 | 0.049 | 0.5494 | 0.4409 |
| trtPlay | 0.085 | 0.063 | 0.049 | 0.1782 | 0.0821 |
| phaseWalking:trtPet | -0.005 | 0.089 | 0.069 | 0.9523 | 0.9386 |
| phaseWalking:trtPlay | -0.013 | 0.089 | 0.069 | 0.8870 | 0.8549 |

Movement:

| term | estimate | std. error (fixed) | std. error (random) | p-value (fixed) | p-value (random) |
|---|---|---|---|---|---|
| intercept | 0.434 | 0.048 | 0.048 | 0.0000 | 0.0000 |
| phaseWalking | 0.221 | 0.068 | 0.061 | 0.0014 | 0.0003 |
| trtPet | -0.104 | 0.068 | 0.061 | 0.1265 | 0.0902 |
| trtPlay | -0.096 | 0.068 | 0.061 | 0.1572 | 0.1171 |
| phaseWalking:trtPet | 0.157 | 0.096 | 0.087 | 0.1020 | 0.0696 |
| phaseWalking:trtPlay | 0.129 | 0.096 | 0.087 | 0.1805 | 0.1380 |

Here is a summary of the results from these tables:

- Proximity to humans is not affected by either treatment or phase (whether human is still or walking).
- Eye contact and movement are influenced only by phase, not by treatment. Exploration is similar, except there is some evidence that this is also affected by treatment: playing with a dog reduces exploration time compared with ignoring it.
- Direction is likely affected not just by the owner walking, but by its interaction with both playing and petting (i.e., the difference is smaller between owner moving and standing still when the dog is ignored than when it isn't), although the evidence for the interaction of phase and petting is weak.
- These results are also fully consistent with a visual analysis of Figure 1.
- Moreover, the outcomes of the random-effect models are aligned with the fixed-effect ones, lending further credibility to the observed outcomes.

Before accepting these conclusions however, we generate diagnostic plots to check whether the models violate the assumptions of Gaussian regression. This is very important to do, because the response variables are proportions between 0 and 1, and therefore strictly speaking the residuals cannot be normally distributed. Below we create four-plot diagnostics for each fixed-effect model:

```
regrDatA |>
  # Generate diagnostic plots and save them as part of the table:
  mutate(dia = map(fixedEffects, \(f) {
    autoplot(f, smooth.colour = NA, alpha = 0.3, colour = "steelblue") +
      theme_bw(base_size = 8)
  } )) |>
  # Add the behaviour as a title to each diagnostic plot:
  mutate(dia = map2(dia, behaviour, \(dia, m) {
    gridExtra::grid.arrange(grobs = dia@plots, top = m)
  } )) |>
  # Loop through each diagnostic and display them:
  mutate(dia = walk(dia, show)) |>
  # Suppress on-screen messages from the output:
  suppressMessages() |>
  capture.output() |>
  invisible()
```

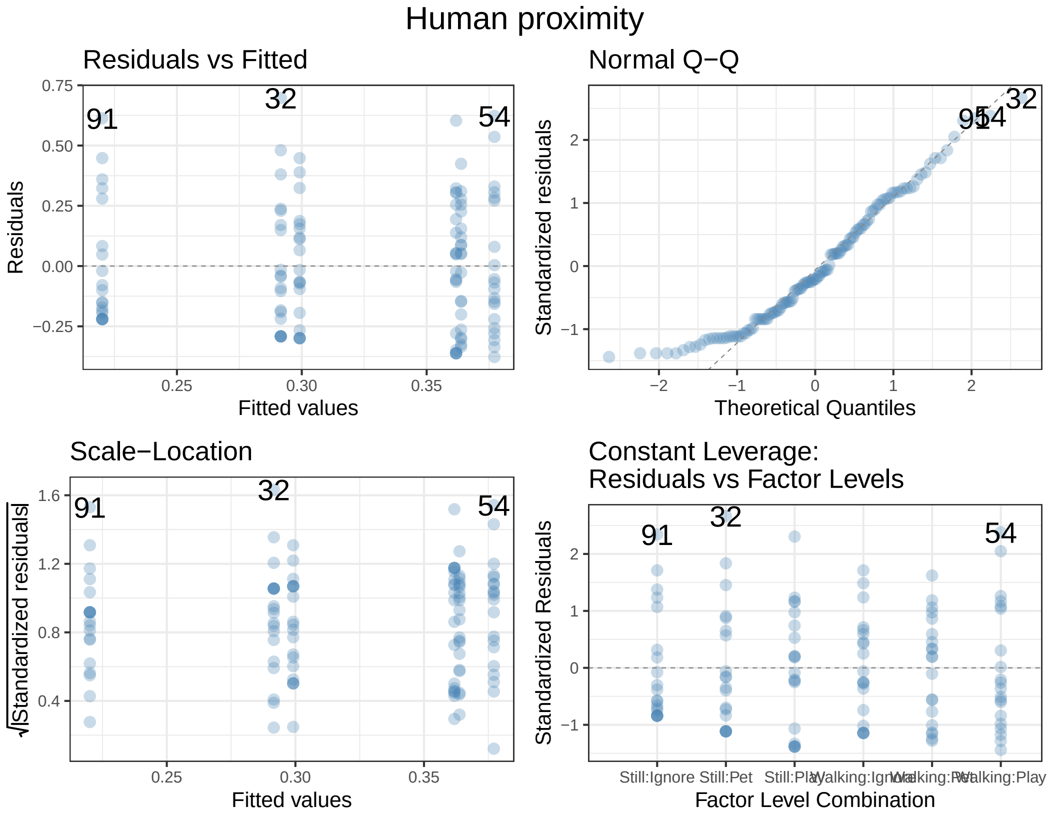
Human proximity
Residuals vs Fitted
Residuals
Fitted values
Normal Q−Q
Standardized residuals
Theoretical Quantiles
Scale−Location
√|Standardized residuals|
Fitted values
Constant Leverage:
Residuals vs Factor Levels
Standardized Residuals
Still:Ignore Still:Pet Still:Play Walking:Ignore Walking:Pet Walking:Play
Factor Level Combination

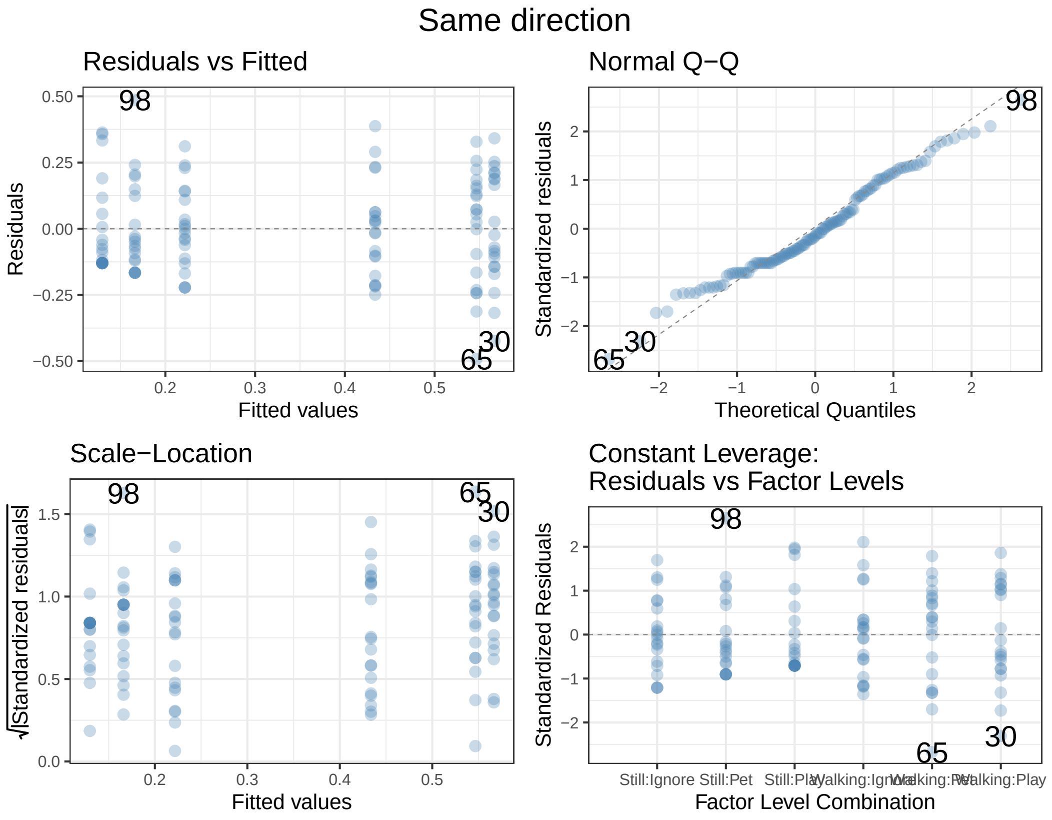
Same direction
Residuals vs Fitted
Residuals
Fitted values
Normal Q−Q
Standardized residuals
Theoretical Quantiles
Scale−Location
√|Standardized residuals|
Fitted values
Constant Leverage:
Residuals vs Factor Levels
Standardized Residuals
Still:Ignore Still:Pet Still:Play Walking:Ignore Walking:Pet Walking:Play
Factor Level Combination

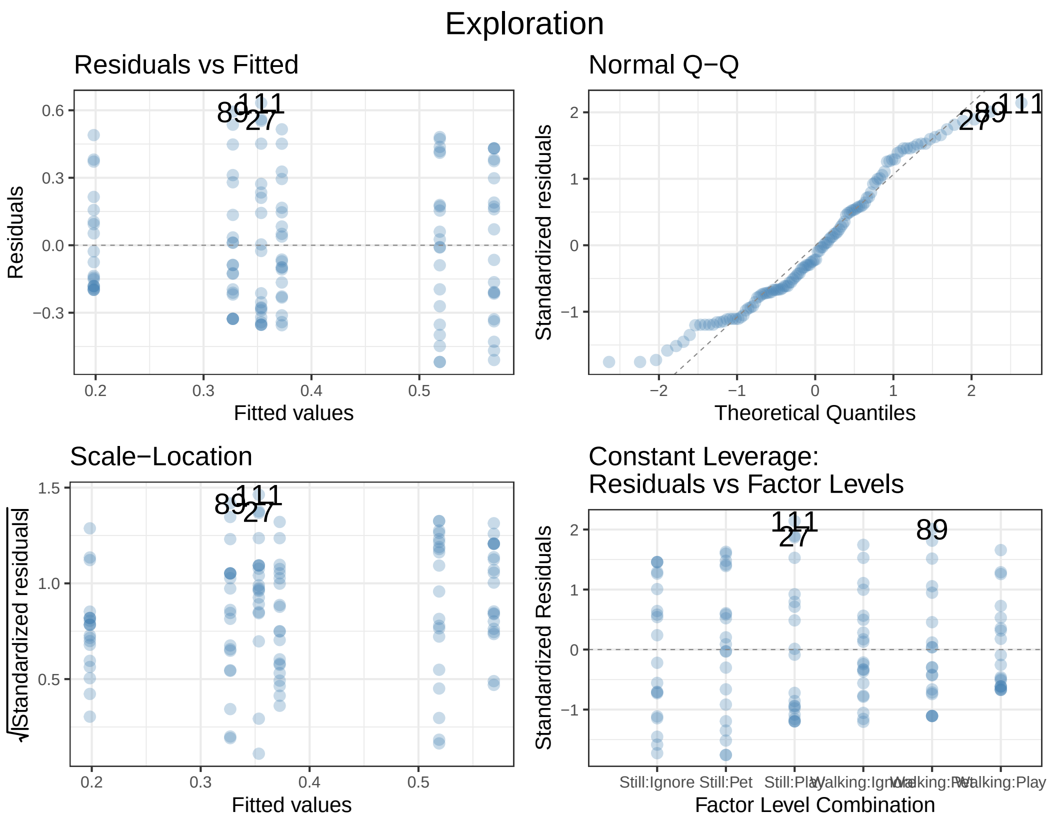
Exploration
Residuals vs Fitted
Residuals
Fitted values
Normal Q−Q
Standardized residuals
Theoretical Quantiles
Scale−Location
√|Standardized residuals|
Fitted values
Constant Leverage:
Residuals vs Factor Levels
Standardized Residuals
Still:Ignore
Still:Pet
Still:Play
Walking:Ignore
Walking:Pet
Walking:Play
Factor Level Combination

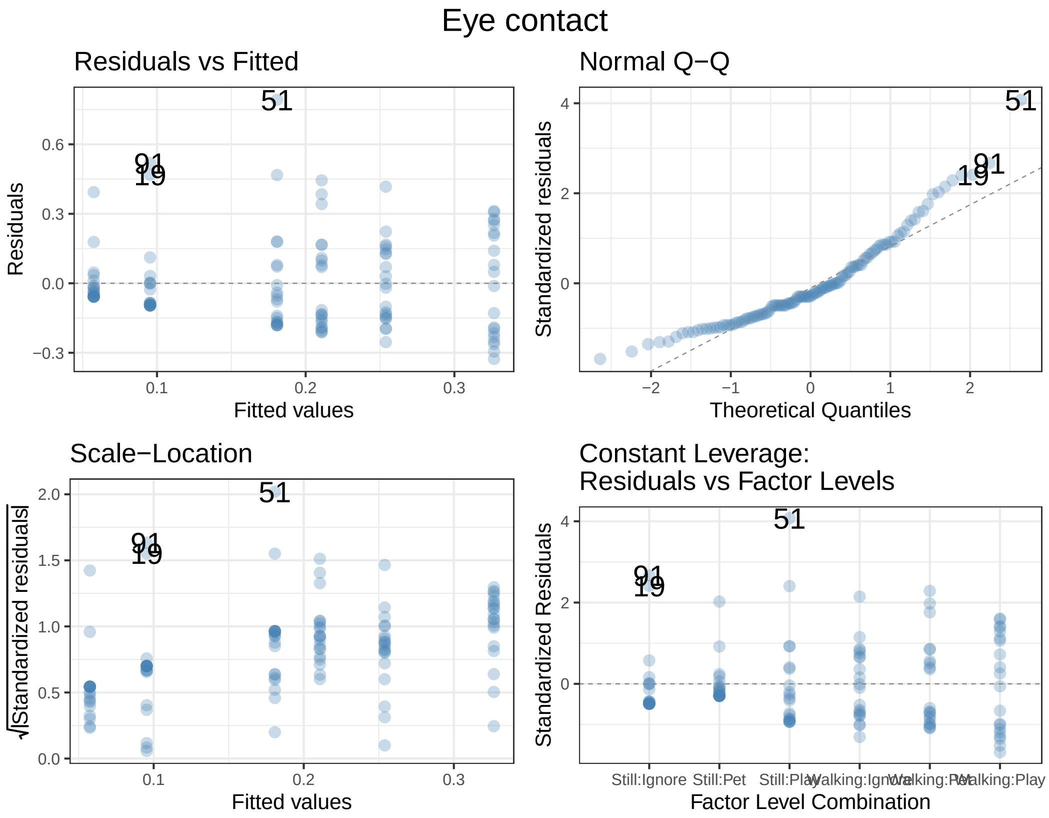
Eye contact
Residuals vs Fitted
Residuals
Fitted values
Normal Q−Q
Standardized residuals
Theoretical Quantiles
Scale−Location
√|Standardized residuals|
Fitted values
Constant Leverage:
Residuals vs Factor Levels
Standardized Residuals
Still:Ignore
Still:Pet
Still:Play
Walking:Ignore
Walking:Pet
Walking:Play
Factor Level Combination

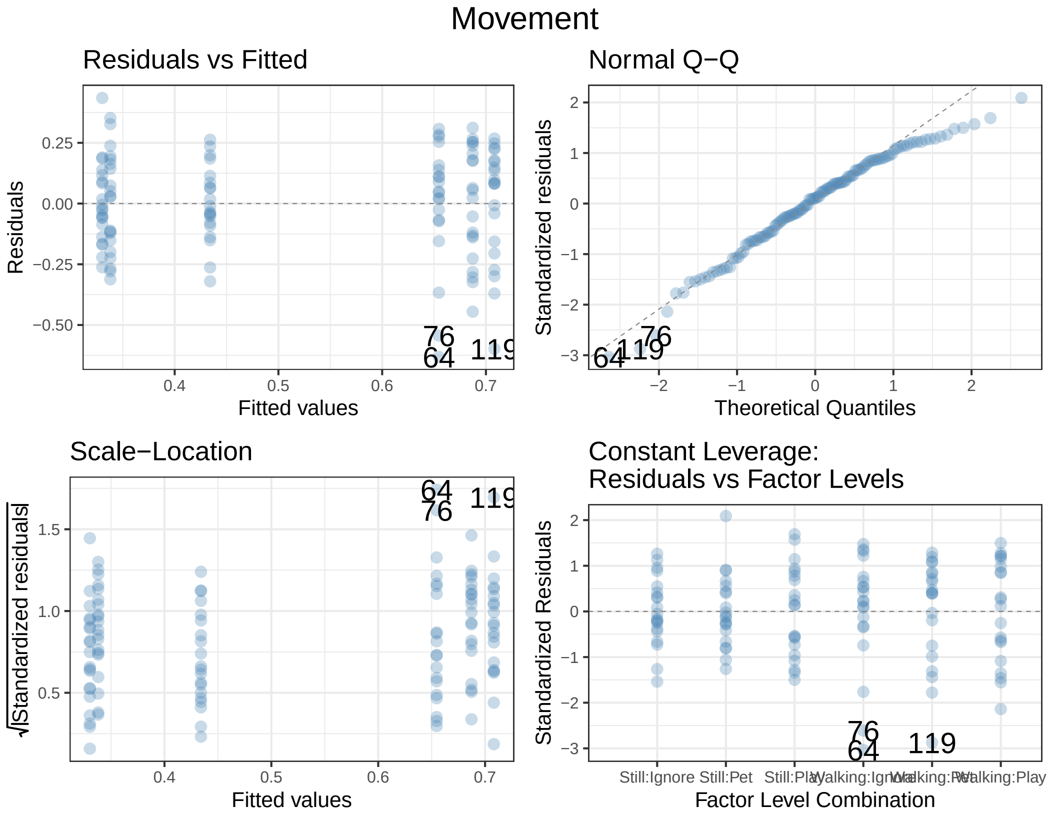


The only problematic aspect of these diagnostic plots is that the normal quantile-quantile graphs occasionally show a relatively large deviation from the expected pattern. This is the case e.g. for eye contact, where small residuals are overestimated and large ones are underestimated. This is to be expected, because the data are confined in the 0-to-1 range, whereas the Gaussian model's domain is the whole real line. A corollary is that the error structure is likely not captured accurately by the model. Since our focus is not on this structure *per se*, this is not a big problem, especially that the assumptions of Gaussian regression are otherwise well satisfied. That said, the regression results for "Eye contact" ought to be treated with caution.

Finally, we can also look at the ANOVA tables of the fixed-effect models from each behaviour. Our experimental design is balanced, with exactly 20 observations per each unique combination of treatment and phase, for each behaviour. Therefore, it does not matter whether one uses type-I (sequential) sum of squares, or type-II or type-III ones; they all give the same answer. For simplicity, we use the default (type-I) provided by the `anova` function:

```
regrDatA |>
  # Extract ANOVA table:
  mutate(linearModel = map(fixedEffects, compose(broom::tidy, anova))) |>
  # Reshape table to human-readable form:
  mutate(linearModel = map(linearModel, \(x) {
    select(x, term, p.value) |>
      drop_na()
  } )) |>
  unnest(linearModel) |>
  mutate(p.value = round(p.value, 4)) |>
```

```
  select(behaviour, term, `p-value` = p.value) |>
  # Easier visual detection of significant results:
  mutate(significance = ifelse(`p-value` < 0.05, "*", "")) |>
  knitr::kable()
```

| behaviour | term | p-value | significance |
|---|---|---|---|
| Human proximity | phase | 0.2596 | |
| Human proximity | trt | 0.1862 | |
| Human proximity | phase:trt | 0.8442 | |
| Same direction | phase | 0.0000 | * |
| Same direction | trt | 0.7838 | |
| Same direction | phase:trt | 0.0240 | * |
| Exploration | phase | 0.0014 | * |
| Exploration | trt | 0.0132 | * |
| Exploration | phase:trt | 0.9456 | |
| Eye contact | phase | 0.0001 | * |
| Eye contact | trt | 0.0272 | * |
| Eye contact | phase:trt | 0.9898 | |
| Movement | phase | 0.0000 | * |
| Movement | trt | 0.7814 | |
| Movement | phase:trt | 0.2185 | |

The "significance" column is a visual helper, with an asterisk if the p-value in the given row is below 0.05.

### 1.4 A non-parametric alternative: the Scheirer–Ray–Hare test

In addition to the linear regressions above, we also perform a Scheirer–Ray–Hare test,[4] which can be thought of as a non-parametric analogue of a two-way ANOVA (in the same manner as the Kruskal–Wallis test is the non-parametric analogue of a one-way ANOVA). We then compare the output of this test with the ANOVA table above. As seen below, the results are qualitatively identical, further indicating that the obtained results are reliable:

```
regrDatA |>
  # Perform Scheirer-Ray-Hare test for each behaviour:
  mutate(scheirerRayHare = map(data, \(x) {
    rcompanion::scheirerRayHare(fraction ~ phase * trt,
                                data = x, verbose = FALSE)
  } )) |>
  # More human-readable tables:
  mutate(scheirerRayHare = map(scheirerRayHare, \(x) {
    mutate(x, term = rownames(x), .before = 1) |>
      filter(term != "Residuals")
  } )) |>
```

[4]Scheirer, J., Ray, W. S., and Hare, N. (1976). The Analysis of Ranked Data Derived from Completely Randomized Factorial Designs. *Biometrics* 32, 429-434

```r
  unnest(scheirerRayHare) |>
  mutate(p.value = round(p.value, 4)) |>
  select(behaviour, term, `p-value` = p.value) |>
  # Easier visual detection of significant results:
  mutate(significance = ifelse(`p-value` < 0.05, "*", "")) |>
  knitr::kable()
```

| behaviour | term | p-value | significance |
|---|---|---|---|
| Human proximity | phase | 0.1496 | |
| Human proximity | trt | 0.1958 | |
| Human proximity | phase:trt | 0.7832 | |
| Same direction | phase | 0.0000 | * |
| Same direction | trt | 0.9776 | |
| Same direction | phase:trt | 0.1453 | |
| Exploration | phase | 0.0043 | * |
| Exploration | trt | 0.0076 | * |
| Exploration | phase:trt | 0.9495 | |
| Eye contact | phase | 0.0000 | * |
| Eye contact | trt | 0.0348 | * |
| Eye contact | phase:trt | 0.7038 | |
| Movement | phase | 0.0000 | * |
| Movement | trt | 0.8190 | |
| Movement | phase:trt | 0.4126 | |

The only difference is in the somewhat contentious interaction between phase and treatment for "Same direction", which comes out nonsignificant here ($p = 0.145$) but was significant in the ANOVA ($p = 0.024$).

## 1.5 Some further analyses

The linear models of Section 1.3 did not take the zero-inflation of the data into account (which can be seen in Figure 1). Fortunately, there is a way to subsume the zero-inflation into the intercept of the model. This is done by the `glmmTMB` function (from the R package of the same name; standing for "Generalized linear mixed model with Template Model Builder"). This function has the `ziformula` option. Setting this to `~1` handles the zero-inflation. Let us fit the models again with this option, for each response variable:

```r
regrDatAZeroinfl <- regrDatA |>
  mutate(zinfl = map(data, \(x)
                     glmmTMB::glmmTMB(fraction ~ phase * trt,
                                      data = x, ziformula = ~1)))

regrDatAZeroinfl |>
  # Create sregression tables:
  mutate(summary = map(zinfl, summary)) |>
  # Loop through the tables to display them:
  mutate(walk2(behaviour, summary, \(m, s) {
```

```r
    cat(str_c(m, ":\n\n")) # Behaviour name
    print(s) # The corresponding table
    cat("\n\n")
  } )) |>
  # Avoid using the resulting table as the output:
  invisible()
```

```
Human proximity:

 Family: gaussian  ( identity )
Formula:          fraction ~ phase * trt
Zero inflation:            ~1
Data: x

     AIC      BIC   logLik deviance df.resid
    34.8     57.1     -9.4     18.8      112


Dispersion estimate for gaussian family (sigma^2): 0.0685

Conditional model:
                     Estimate Std. Error z value Pr(>|z|)
(Intercept)           0.22010    0.05851   3.762 0.000169 ***
phaseWalking          0.07905    0.08275   0.955 0.339418
trtPet                0.07150    0.08275   0.864 0.387547
trtPlay               0.14170    0.08275   1.712 0.086816 .
phaseWalking:trtPet  -0.00685    0.11702  -0.059 0.953322
phaseWalking:trtPlay -0.06370    0.11702  -0.544 0.586208
---
Signif. codes:  0 '***' 0.001 '**' 0.01 '*' 0.05 '.' 0.1 ' ' 1

Zero-inflation model:
            Estimate Std. Error z value Pr(>|z|)
(Intercept)    -25.2    30389.8  -0.001    0.999


Same direction:

 Family: gaussian  ( identity )
Formula:          fraction ~ phase * trt
Zero inflation:            ~1
Data: x

     AIC      BIC   logLik deviance df.resid
   -50.1    -27.8     33.0    -66.1      112
```

```
Dispersion estimate for gaussian family (sigma^2): 0.0338

Conditional model:
                     Estimate Std. Error z value Pr(>|z|)
(Intercept)           0.22175    0.04108   5.398 6.75e-08 ***
phaseWalking          0.21200    0.05810   3.649 0.000263 ***
trtPet               -0.05565    0.05810  -0.958 0.338142
trtPlay              -0.09205    0.05810  -1.584 0.113112
phaseWalking:trtPet   0.16850    0.08216   2.051 0.040291 *
phaseWalking:trtPlay  0.22490    0.08216   2.737 0.006197 **
---
Signif. codes:  0 '***' 0.001 '**' 0.01 '*' 0.05 '.' 0.1 ' ' 1

Zero-inflation model:
            Estimate Std. Error z value Pr(>|z|)
(Intercept)   -23.98   15443.10  -0.002    0.999
```

Exploration:

```
 Family: gaussian  ( identity )
Formula:          fraction ~ phase * trt
Zero inflation:            ~1
Data: x

     AIC      BIC   logLik deviance df.resid
    64.0     86.3    -24.0     48.0      112


Dispersion estimate for gaussian family (sigma^2): 0.0873

Conditional model:
                     Estimate Std. Error z value Pr(>|z|)
(Intercept)           0.56920    0.06608   8.614   <2e-16 ***
phaseWalking         -0.19665    0.09345  -2.104   0.0353 *
trtPet               -0.05025    0.09345  -0.538   0.5907
trtPlay              -0.21580    0.09345  -2.309   0.0209 *
phaseWalking:trtPet   0.00485    0.13215   0.037   0.9707
phaseWalking:trtPlay  0.04150    0.13215   0.314   0.7535
---
Signif. codes:  0 '***' 0.001 '**' 0.01 '*' 0.05 '.' 0.1 ' ' 1

Zero-inflation model:
            Estimate Std. Error z value Pr(>|z|)
(Intercept)   -24.52   21076.68  -0.001    0.999
```

Eye contact:

```
 Family: gaussian  ( identity )
Formula:          fraction ~ phase * trt
Zero inflation:            ~1
Data: x

     AIC      BIC   logLik deviance df.resid
   -36.7    -14.4     26.3    -52.7      112


Dispersion estimate for gaussian family (sigma^2): 0.0378

Conditional model:
                     Estimate Std. Error z value Pr(>|z|)
(Intercept)           0.09535    0.04345   2.195  0.02819 *
phaseWalking          0.15860    0.06144   2.581  0.00985 **
trtPet               -0.03785    0.06144  -0.616  0.53790
trtPlay               0.08540    0.06144   1.390  0.16457
phaseWalking:trtPet  -0.00535    0.08690  -0.062  0.95091
phaseWalking:trtPlay -0.01270    0.08690  -0.146  0.88380
---
Signif. codes:  0 '***' 0.001 '**' 0.01 '*' 0.05 '.' 0.1 ' ' 1

Zero-inflation model:
            Estimate Std. Error z value Pr(>|z|)
(Intercept)   -24.43   19829.93  -0.001    0.999


Movement:

 Family: gaussian  ( identity )
Formula:          fraction ~ phase * trt
Zero inflation:            ~1
Data: x

     AIC      BIC   logLik deviance df.resid
   -20.1      2.2     18.1    -36.1      112


Dispersion estimate for gaussian family (sigma^2): 0.0433

Conditional model:
                     Estimate Std. Error z value Pr(>|z|)
(Intercept)           0.43415    0.04654   9.328  < 2e-16 ***
phaseWalking          0.22055    0.06582   3.351 0.000806 ***
trtPet               -0.10395    0.06582  -1.579 0.114274
trtPlay              -0.09615    0.06582  -1.461 0.144079
phaseWalking:trtPet   0.15745    0.09309   1.691 0.090751 .
```

```
phaseWalking:trtPlay  0.12870    0.09309   1.383 0.166789
---
Signif. codes:  0 '***' 0.001 '**' 0.01 '*' 0.05 '.' 0.1 ' ' 1

Zero-inflation model:
            Estimate Std. Error z value Pr(>|z|)
(Intercept)   -24.95   23841.62  -0.001    0.999
```

Comparing the regression results with- and without the handling of zero-inflation reveals that the predictions of the corresponding models are qualitatively identical. However, it is instructive to compare their AIC scores:

```
regrDatAZeroinfl |>
  transmute(behaviour,
            `AIC (original)` = map_dbl(fixedEffects, AIC),
            `AIC (zero-inflated)` = map_dbl(zinfl, AIC)) |>
  knitr::kable()
```

| behaviour | AIC (original) | AIC (zero-inflated) |
|---|---:|---:|
| Human proximity | 32.78449 | 34.78449 |
| Same direction | -52.08910 | -50.08910 |
| Exploration | 61.96347 | 63.96347 |
| Eye contact | -38.65096 | -36.65096 |
| Movement | -22.13834 | -20.13834 |

As seen, the AIC score is always lower (better) by exactly 2 for the original model than the one that handles zero-inflation—which, by the meaning of AIC,[5] implies that all this has done is introduce one extra parameter without any gains in log likelihood. So incorporating the handling of zero-inflation leads to inferior models which predicts the same things the simpler models do anyway.

Another possibility is to perform zero-inflated Gamma or (quasi-)binomial regression instead of a Gaussian one. Without going into more detailed analysis, here are some results which demonstrate that they give poor results compared with the Gaussian model fits (compare the AIC scores with those in the table above):

```
studyA |>
  filter(behaviour == "Movement") |>
  glmmTMB::glmmTMB(fraction ~ phase * treatment, data = _,
                   family = Gamma, ziformula = ~1) |>
  summary()
```

```
 Family: Gamma  ( inverse )
Formula:          fraction ~ phase * treatment
Zero inflation:            ~1
Data: filter(studyA, behaviour == "Movement")
```

[5]By definition, $\text{AIC} = 2k - 2\log(L)$, where $k$ is the number of parameters in the model and $L$ is the maximized likelihood function.

```
     AIC      BIC   logLik deviance df.resid 
    11.1     33.4      2.5     -4.9      112 


Dispersion estimate for Gamma family (sigma^2): 0.275 

Conditional model:
                           Estimate Std. Error z value Pr(>|z|)    
(Intercept)                  2.3034     0.2699   8.535   <2e-16 ***
phaseWalking                -0.7759     0.3238  -2.396   0.0166 *  
treatmentPet                 0.7251     0.4458   1.627   0.1038    
treatmentPlay                0.6552     0.4393   1.492   0.1358    
phaseWalking:treatmentPet   -0.8405     0.5080  -1.654   0.0980 .  
phaseWalking:treatmentPlay  -0.7276     0.5040  -1.443   0.1489    
---
Signif. codes:  0 '***' 0.001 '**' 0.01 '*' 0.05 '.' 0.1 ' ' 1

Zero-inflation model:
            Estimate Std. Error z value Pr(>|z|)
(Intercept)   -26.96   65423.61       0        1
```

```r
studyA |>
  filter(behaviour == "Movement") |>
  glmmTMB::glmmTMB(fraction ~ phase * treatment, data = _,
                   family = binomial, ziformula = ~1) |>
  summary()
```

```
 Family: binomial  ( logit )
Formula:          fraction ~ phase * treatment
Zero inflation:            ~1
Data: filter(studyA, behaviour == "Movement")

     AIC      BIC   logLik deviance df.resid 
   167.1    186.6    -76.6    153.1      113 


Conditional model:
                           Estimate Std. Error z value Pr(>|z|)
(Intercept)                 -0.2649     0.4511  -0.587    0.557
phaseWalking                 0.9047     0.6517   1.388    0.165
treatmentPet                -0.4423     0.6554  -0.675    0.500
treatmentPlay               -0.4073     0.6534  -0.623    0.533
phaseWalking:treatmentPet    0.6892     0.9448   0.730    0.466
phaseWalking:treatmentPlay   0.5548     0.9385   0.591    0.554

Zero-inflation model:
            Estimate Std. Error z value Pr(>|z|)
(Intercept)   -23.21   10018.41  -0.002    0.998
```

## 2 Study B

We first load and clean the data:

```
studyB <- read_csv("study-B.csv") |>
  # Drop unnecessary columns:
  select(!ends_with("sex") & !dogage) |>
  # Capitalized entries in `phase` and `group`:
  mutate(across(c(phase, group), str_to_title)) |>
  mutate(group = fct_relevel(group, "Companion", "Hunting")) |>
  # Arrange measured behaviours in key-value pairs:
  pivot_longer(cols = humanprox:outview,
               names_to = "behaviour",
               values_to = "fraction") |>
  # Human-readable names for the various dog behaviours:
  mutate(behaviour = case_match(
    behaviour,
    "humanprox" ~ "Human proximity",
    "direction" ~ "Same direction",
    "exploration" ~ "Exploration",
    "eyecon" ~ "Eye contact",
    "move" ~ "Movement",
    "outview" ~ "Out of view"
  ))
```

Here are the first few rows of this cleaned table:

```
studyB |>
  head(n = 12) |>
  knitr::kable()
```

| id | phase | group | behaviour | fraction |
|---|---|---|---|---|
| 1 | Still | Ancient | Human proximity | 0.028 |
| 1 | Still | Ancient | Same direction | 0.071 |
| 1 | Still | Ancient | Exploration | 0.000 |
| 1 | Still | Ancient | Eye contact | 0.000 |
| 1 | Still | Ancient | Movement | 0.399 |
| 1 | Still | Ancient | Out of view | 0.000 |
| 1 | Walking | Ancient | Human proximity | 0.048 |
| 1 | Walking | Ancient | Same direction | 0.058 |
| 1 | Walking | Ancient | Exploration | 0.026 |
| 1 | Walking | Ancient | Eye contact | 0.269 |
| 1 | Walking | Ancient | Movement | 0.163 |
| 1 | Walking | Ancient | Out of view | 0.000 |

We can now visualize these data (Figure 3):

```
studyB |>
  plotRawData(column = group, label = "Breed type")
```

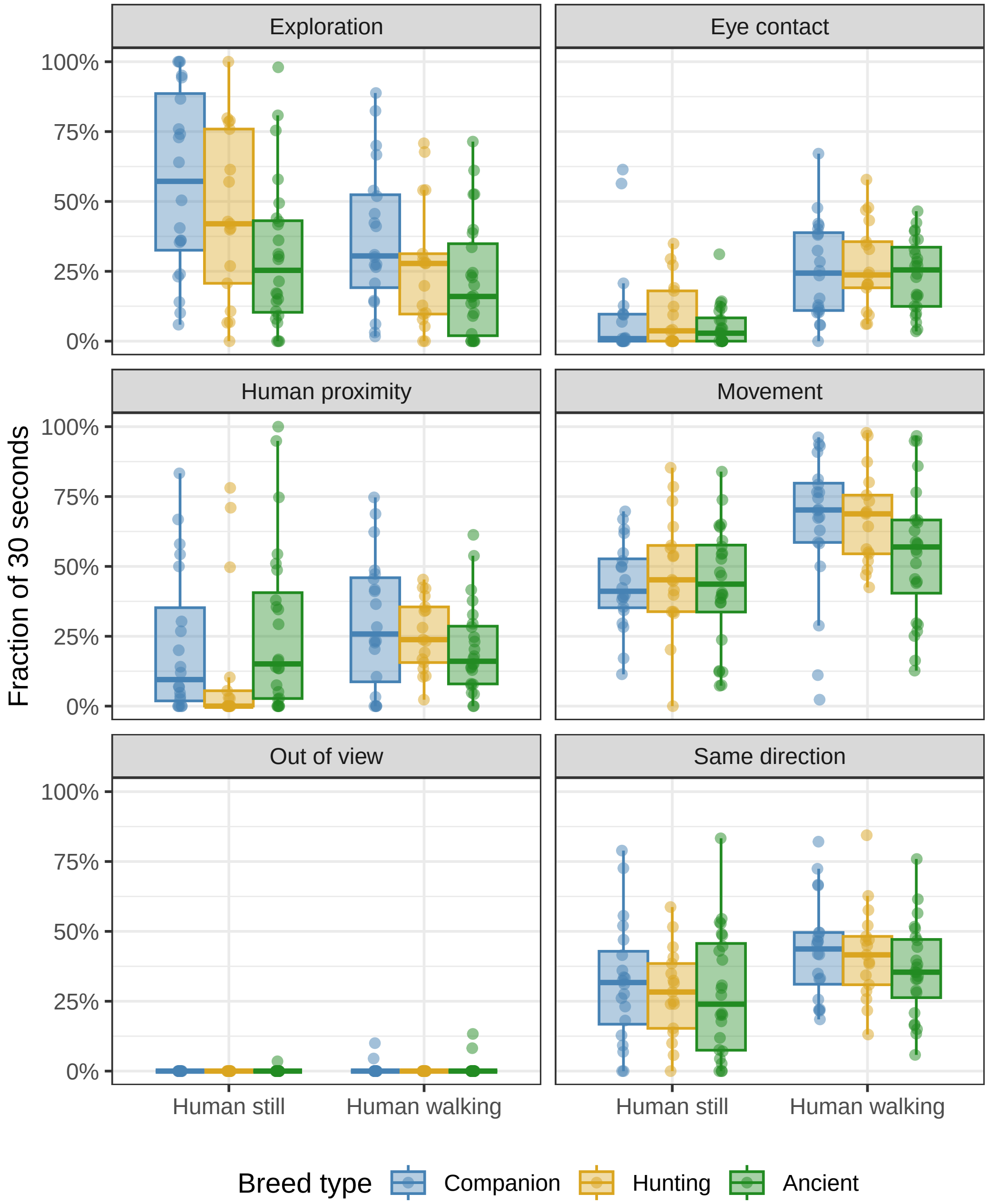


Figure 3: The fraction of time out of the total 30 seconds (y-axis) spent by dogs on each of six behaviours (panel labels). The dogs were either companion dogs, hunting dogs, or from an ancient breed (colours). The owner was either standing still or walking (x-axis). Each point corresponds to one dog's measurement; the box plots summarize these points.

As in Study A (Section 1), the data for being out-of-view are mostly zeros and were excluded from subsequent analyses.

Interpreting the data in Figure 3 is simpler than it was in Study A, because of the lack of repeated

measures. We fit a linear model for each behaviour with phase, group, and their interaction as predictors. Additionally, we also perform the non-parametric Scheirer–Ray–Hare test and compare its output with that of the linear regression model.

```
regrDatB <- studyB |>
  # Remove the undesired out-of-view behaviour:
  filter(behaviour != "Out of view") |>
  # Create a sub-table of the data for each behaviour:
  nest(data = !behaviour) |>
  # Fit linear model for each behaviour:
  mutate(linearModel = map(data, \(x) {
    lm(fraction ~ phase * group, data = x)
  } )) |>
  # Perform Scheirer-Ray-Hare test for each behaviour:
  mutate(scheirerRayHare = map(data, \(x) {
    rcompanion::scheirerRayHare(fraction ~ phase * group,
                                data = x, verbose = FALSE)
  } ))
```

From this, we generate the regression tables:

```
regrDatB |>
  mutate(linearModel = map(linearModel, broom::tidy)) |>
  mutate(linearModel = map(linearModel, \(x) {
    select(x, !statistic) |>
      rename(`std. error` = std.error, `p-value` = p.value) |>
      mutate(term = ifelse(term == "(Intercept)", "intercept", term)) |>
      mutate(across(!term, \(col) round(col, 4)))
  } )) |>
  # Loop through each joint table and display them:
  mutate(walk2(behaviour, linearModel, \(m, tab) {
    cat(str_c(m, ":")) # Behaviour name
    print(knitr::kable(tab)) # The corresponding table
  } )) |>
  # Avoid using the resulting table as the output:
  invisible()
```

Human proximity:

| term | estimate | std. error | p-value |
|---|---|---|---|
| intercept | 0.2201 | 0.0525 | 0.0001 |
| phaseWalking | 0.0791 | 0.0743 | 0.2893 |
| groupHunting | -0.0905 | 0.0775 | 0.2453 |
| groupAncient | 0.0519 | 0.0711 | 0.4669 |
| phaseWalking:groupHunting | 0.0483 | 0.1096 | 0.6601 |
| phaseWalking:groupAncient | -0.1464 | 0.1005 | 0.1480 |

Same direction:

| term | estimate | std. error | p-value |
|---|---|---|---|
| intercept | 0.3188 | 0.0425 | 0.0000 |
| phaseWalking | 0.1149 | 0.0600 | 0.0581 |
| groupHunting | -0.0371 | 0.0626 | 0.5543 |
| groupAncient | -0.0401 | 0.0575 | 0.4873 |
| phaseWalking:groupHunting | 0.0251 | 0.0886 | 0.7774 |
| phaseWalking:groupAncient | -0.0327 | 0.0813 | 0.6884 |

Exploration:

| term | estimate | std. error | p-value |
|---|---|---|---|
| intercept | 0.5692 | 0.0598 | 0.0000 |
| phaseWalking | -0.1966 | 0.0846 | 0.0219 |
| groupHunting | -0.1173 | 0.0883 | 0.1867 |
| groupAncient | -0.2624 | 0.0810 | 0.0016 |
| phaseWalking:groupHunting | 0.0134 | 0.1249 | 0.9150 |
| phaseWalking:groupAncient | 0.1073 | 0.1146 | 0.3512 |

Eye contact:

| term | estimate | std. error | p-value |
|---|---|---|---|
| intercept | 0.0954 | 0.0316 | 0.0032 |
| phaseWalking | 0.1586 | 0.0447 | 0.0006 |
| groupHunting | -0.0023 | 0.0467 | 0.9609 |
| groupAncient | -0.0417 | 0.0428 | 0.3323 |
| phaseWalking:groupHunting | 0.0183 | 0.0660 | 0.7815 |
| phaseWalking:groupAncient | 0.0255 | 0.0606 | 0.6747 |

Movement:

| term | estimate | std. error | p-value |
|---|---|---|---|
| intercept | 0.4341 | 0.0479 | 0.0000 |
| phaseWalking | 0.2206 | 0.0677 | 0.0015 |
| groupHunting | 0.0453 | 0.0707 | 0.5231 |
| groupAncient | -0.0038 | 0.0649 | 0.9532 |
| phaseWalking:groupHunting | -0.0300 | 0.0999 | 0.7648 |
| phaseWalking:groupAncient | -0.1003 | 0.0917 | 0.2764 |

And we can look at the diagnostic plots:

```
regrDatB |>
  # Generate diagnostic plots and save them as part of the table:
  mutate(dia = map(linearModel, \(f) {
```

```
  autoplot(f, smooth.colour = NA, alpha = 0.3, colour = "steelblue") +
    theme_bw(base_size = 8)
} )) |>
# Add the behaviour as a title to each diagnostic plot:
mutate(dia = map2(dia, behaviour, \(dia, m) {
  gridExtra::grid.arrange(grobs = dia@plots, top = m)
} )) |>
# Suppress on-screen messages from the output:
suppressMessages() |>
capture.output() |>
invisible()
```

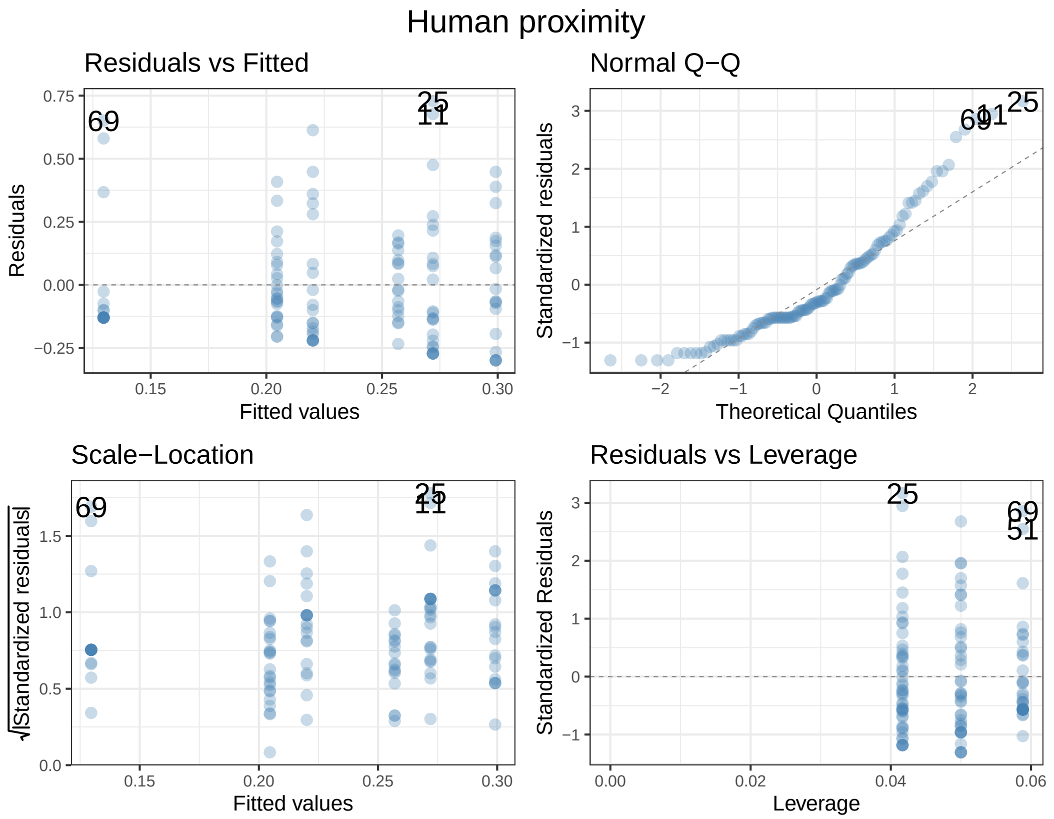

Same direction

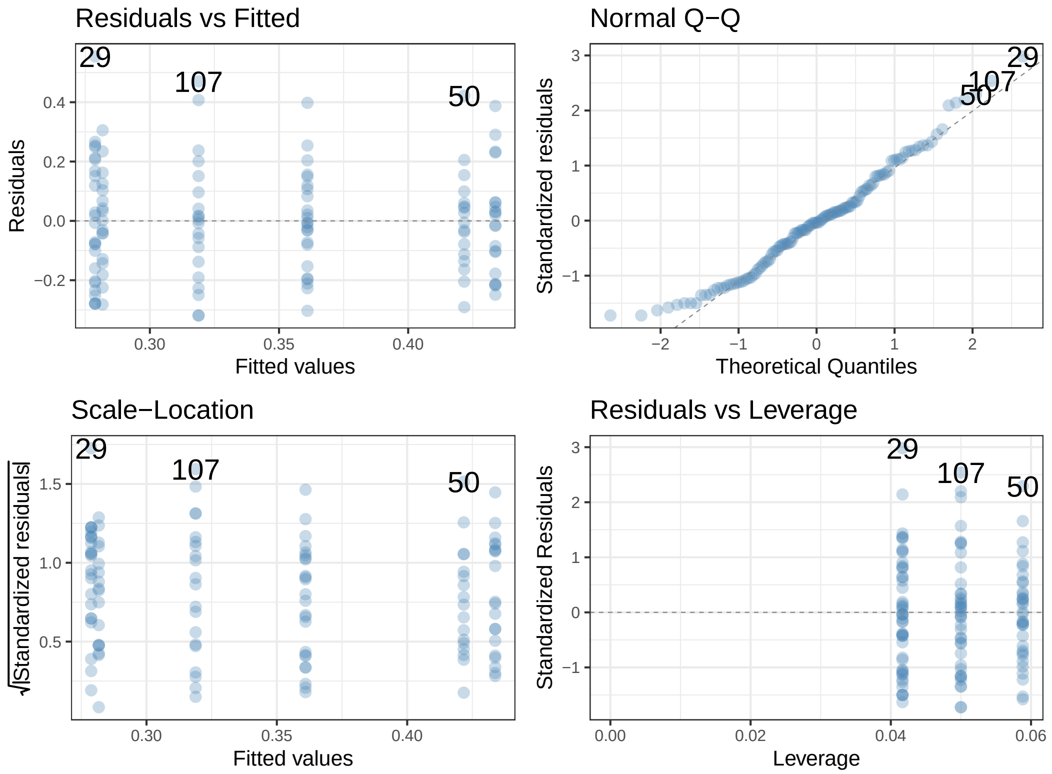


Exploration

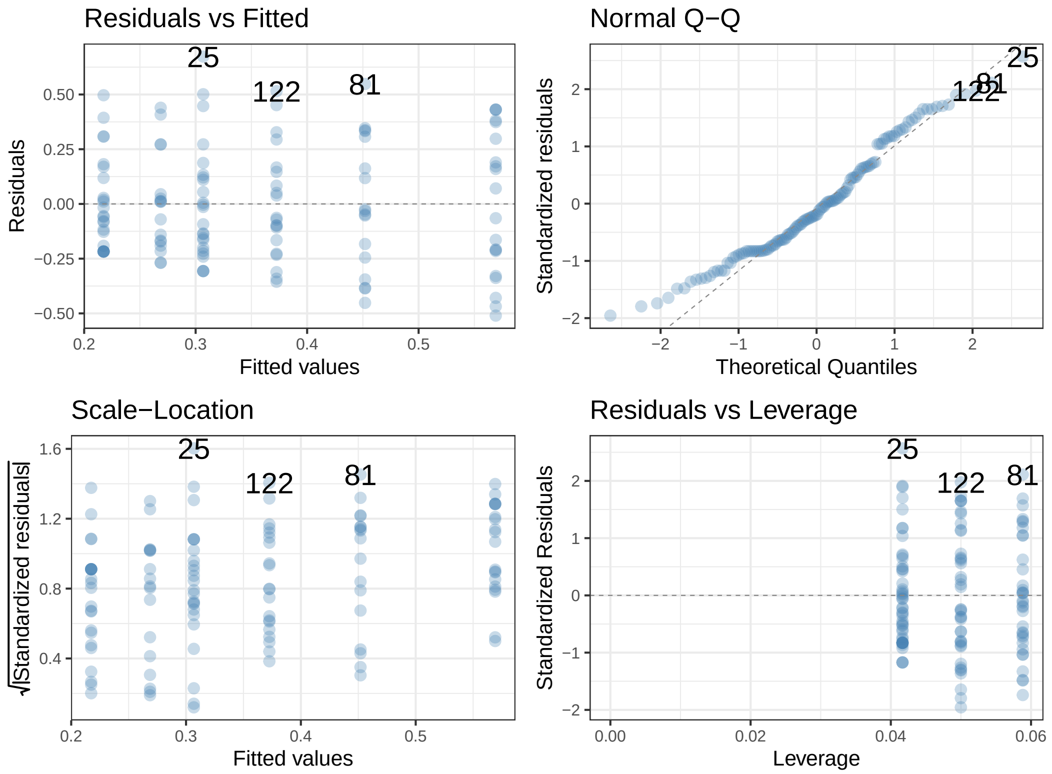

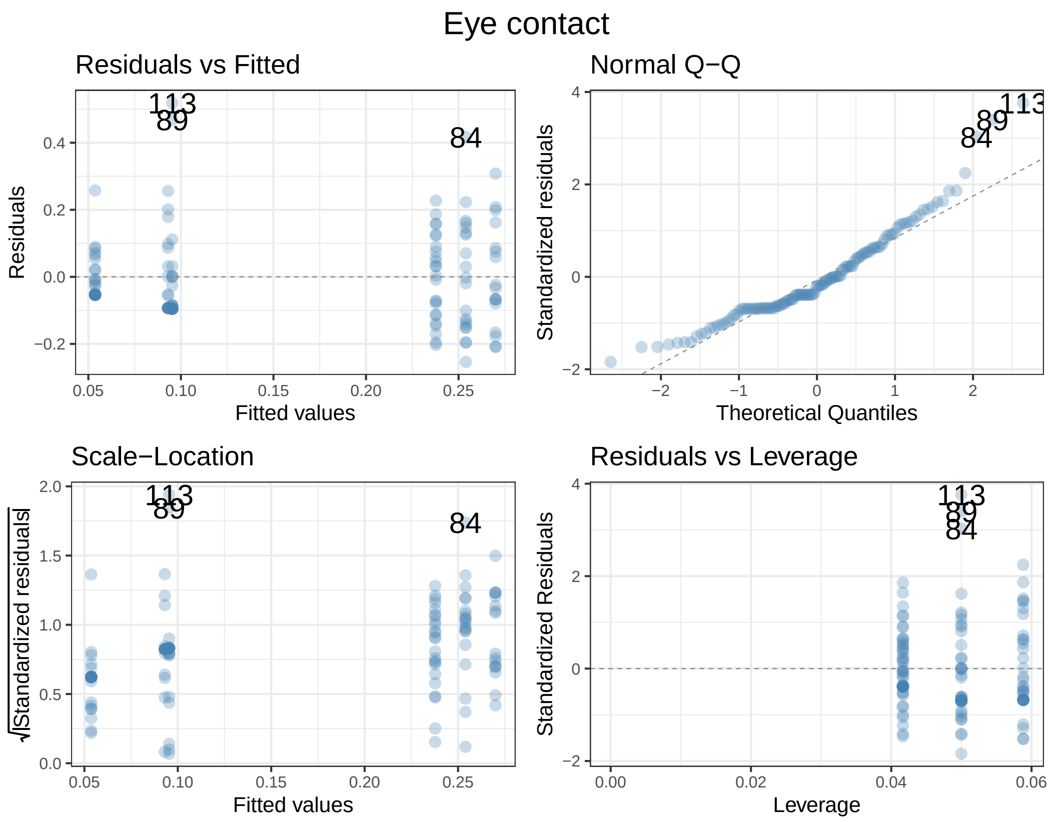
Eye contact
Residuals vs Fitted
Residuals
Fitted values
113
89
84
Normal Q−Q
Standardized residuals
Theoretical Quantiles
113
89
84
Scale−Location
√|Standardized residuals|
Fitted values
113
89
84
Residuals vs Leverage
Standardized Residuals
Leverage
113
89
84

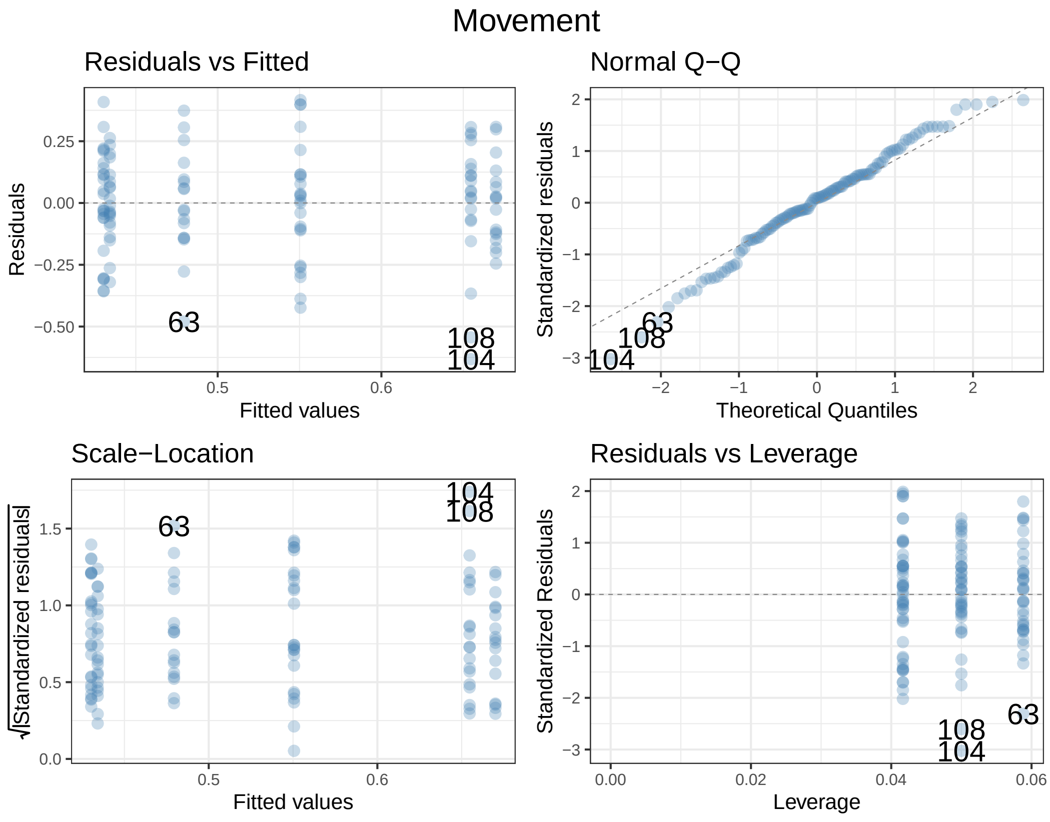
Movement
Residuals vs Fitted
Residuals
Fitted values
63
108
104
Normal Q−Q
Standardized residuals
Theoretical Quantiles
63
108
104
Scale−Location
√|Standardized residuals|
Fitted values
63
104
108
Residuals vs Leverage
Standardized Residuals
Leverage
63
108
104

While the quantile-quantile plots for “Human proximity” and “Eye contact” do not look very good (and therefore their outcomes should be treated with caution), the other behaviours’ diagnostics are acceptable.

We now look at the ANOVA table for each behaviour. Unlike in Study A (Section 1), here the experimental design is not balanced: for each behaviour, there are 20 companion dogs, 17 hunting dogs, and 24 dogs from ancient breeds. We therefore use type-II sum of squares, which is implemented by the `Anova` function of the `car` package:

```
regrDatB |>
  # Extract ANOVA table:
  mutate(linearModel = map(linearModel,
                           \(x) broom::tidy(car::Anova(x, type = 2)))) |>
  # Reshape table to human-readable form:
  mutate(linearModel = map(linearModel, \(x) {
    select(x, term, p.value) |>
      drop_na()
  } )) |>
  unnest(linearModel) |>
  mutate(p.value = round(p.value, 4)) |>
  select(behaviour, term, `p-value` = p.value) |>
  # Easier visual detection of significant results:
  mutate(significance = ifelse(`p-value` < 0.05, "*", "")) |>
  knitr::kable()
```

| behaviour | term | p-value | significance |
|---|---|---|---|
| Human proximity | phase | 0.4134 | |
| Human proximity | group | 0.4725 | |
| Human proximity | phase:group | 0.1436 | |
| Same direction | phase | 0.0019 | * |
| Same direction | group | 0.3801 | |
| Same direction | phase:group | 0.7889 | |
| Exploration | phase | 0.0024 | * |
| Exploration | group | 0.0019 | * |
| Exploration | phase:group | 0.5927 | |
| Eye contact | phase | 0.0000 | * |
| Eye contact | group | 0.4645 | |
| Eye contact | phase:group | 0.9126 | |
| Movement | phase | 0.0000 | * |
| Movement | group | 0.1974 | |
| Movement | phase:group | 0.5297 | |

As in Section 1.3, the last column shows an asterisk if the p-value in the given row is below 0.05.

From the above regression tables, we draw the following conclusions:

- Companion dogs are more explorative than dogs from ancient breeds.
- Human walking positively impacts eye contact, movement, and perhaps same direction. It negatively impacts exploration.

- There is no evidence of any interactions between breed type and phase (i.e., human standing still or walking).

To lend further credibility to the results, we also check the outcome of the non-parametric Scheirer–Ray–Hare tests and see if they are consistent with the linear model.

```r
regrDatB |>
  # More human-readable tables:
  mutate(scheirerRayHare = map(scheirerRayHare, \(x) {
    mutate(x, term = rownames(x), .before = 1) |>
      filter(term != "Residuals")
  } )) |>
  unnest(scheirerRayHare) |>
  mutate(p.value = round(p.value, 4)) |>
  select(behaviour, term, `p-value` = p.value) |>
  # Easier visual detection of significant results:
  mutate(significance = ifelse(`p-value` < 0.05, "*", "")) |>
  knitr::kable()
```

| behaviour | term | p-value | significance |
|---|---|---|---|
| Human proximity | phase | 0.0192 | * |
| Human proximity | group | 0.4508 | |
| Human proximity | phase:group | 0.0605 | |
| Same direction | phase | 0.0019 | * |
| Same direction | group | 0.5083 | |
| Same direction | phase:group | 0.7825 | |
| Exploration | phase | 0.0075 | * |
| Exploration | group | 0.0044 | * |
| Exploration | phase:group | 0.8503 | |
| Eye contact | phase | 0.0000 | * |
| Eye contact | group | 0.7712 | |
| Eye contact | phase:group | 0.9310 | |
| Movement | phase | 0.0000 | * |
| Movement | group | 0.2497 | |
| Movement | phase:group | 0.3428 | |

These results are fully in line with those of the linear model, with one addition: this test believes that the human walking or standing influences proximity, to a degree that is unlikely to be due to chance ($p = 0.019$). Indeed, it is difficult to say based on a visual inspection of Figure 3 whether human movement has an overall effect on proximity (with the exception of dogs from hunting breeds). Despite this point, it is clear that there is an overall qualitative agreement between the linear regression model and the Scheirer–Ray–Hare test.

# 3 Study C

As before, we load and clean the data first:

```
studyC <- read_csv("study-C.csv") |>
  # Drop unnecessary columns:
  select(!animalage) |>
  # Arrange measured behavioural variables in key-value pairs:
  pivot_longer(cols = humanprox:outview,
               names_to = "behaviour",
               values_to = "fraction") |>
  # Capitalized entries in `phase` and `group`:
  mutate(across(c(phase, group), str_to_title)) |>
  # Human-readable names for the various dog behaviours:
  mutate(behaviour = case_match(
    behaviour,
    "humanprox" ~ "Human proximity",
    "direction" ~ "Same direction",
    "exploration" ~ "Exploration",
    "eyecon" ~ "Eye contact",
    "move" ~ "Movement",
    "outview" ~ "Out of view"
  )) |>
  # Remove the two behaviours that were difficult to measure:
  filter(!(behaviour %in% c("Eye contact", "Exploration")))
```

Due to the greater distance and more fences between the camera and the test arena, "Eye contact" and "Exploration" were challenging to assess from the videos. We therefore do not analyze these behaviours and removed them from the data above.

The first few rows of this table are:

```
studyC |>
  head(n = 12) |>
  knitr::kable()
```

| id | phase | group | behaviour | fraction |
|---|---|---|---|---|
| 1 | Still | Dog | Human proximity | 0.000 |
| 1 | Still | Dog | Same direction | 0.515 |
| 1 | Still | Dog | Movement | 0.719 |
| 1 | Still | Dog | Out of view | 0.045 |
| 1 | Walking | Dog | Human proximity | 0.000 |
| 1 | Walking | Dog | Same direction | 0.208 |
| 1 | Walking | Dog | Movement | 1.000 |
| 1 | Walking | Dog | Out of view | 0.359 |
| 2 | Still | Dog | Human proximity | 0.000 |
| 2 | Still | Dog | Same direction | 0.318 |
| 2 | Still | Dog | Movement | 0.869 |
| 2 | Still | Dog | Out of view | 0.021 |

We can visualize these data (Figure 4):

```
# Plot data for all behaviours:
studyC |>
  plotRawData(column = group, label = "Species")
```

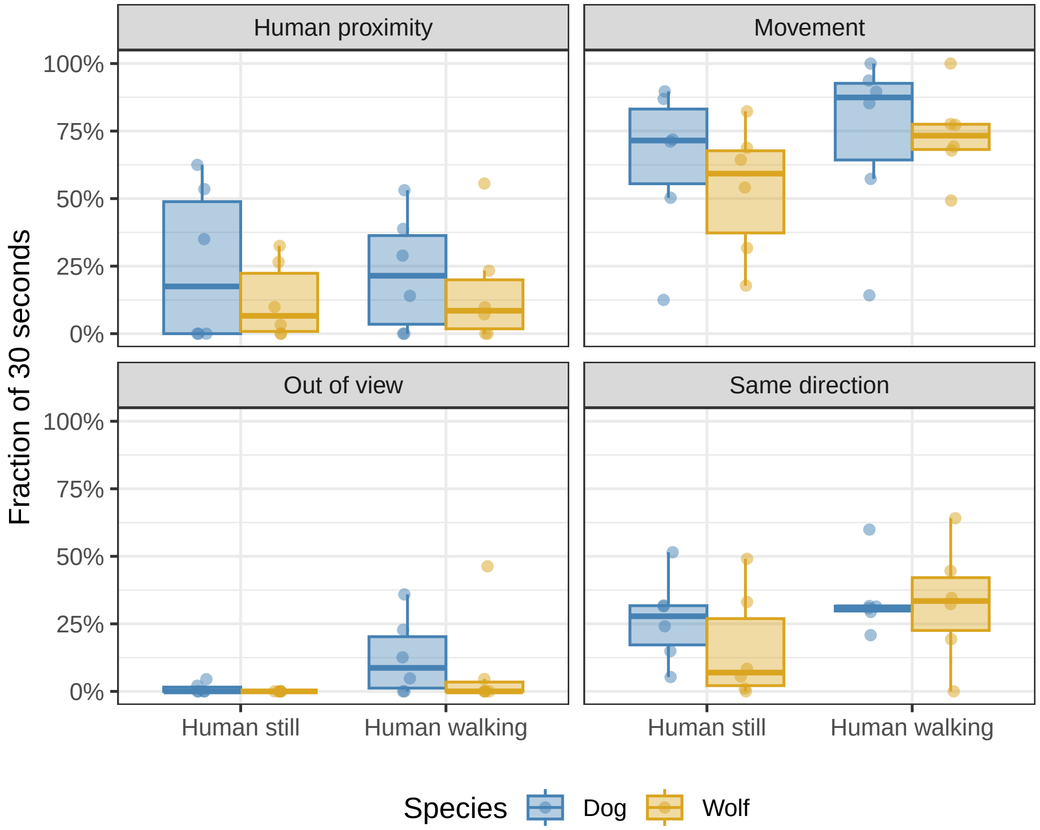


Figure 4: The fraction of time out of the total 30 seconds (y-axis) spent by dogs and wolves (colours) on each of four behaviours (panel labels). A human was either standing still or walking (x-axis). Each point corresponds to one measurement; the box plots summarize these points.

It looks as if there could be a difference between wolves' and dogs' tendencies to be out of view depending on whether a human is standing still or walking. Otherwise, there might unfortunately not be sufficient data available to have strong support for any further conclusions.

To see if that is so, we fit a linear model for each behaviour with phase, group, and their interaction as predictors. We also employ the non-parametric Scheirer–Ray–Hare test.

```
regrDatC <- studyC |>
  # Create a sub-table of the data for each behaviour:
  nest(data = !behaviour) |>
  # Fit linear model with fixed effects for each behaviour:
  mutate(linearModel = map(data, \(x) {
    lm(fraction ~ phase * group, data = x)
  } )) |>
  # Scheirer-Ray-Hare test:
```

```
  mutate(scheirerRayHare = map(data, \(x) {
    rcompanion::scheirerRayHare(fraction ~ phase * group,
                                data = x, verbose = FALSE)
  } ))
```

We can look at the diagnostic plots from the linear regressions:

```
regrDatC |>
  # Generate diagnostic plots and save them as part of the table:
  mutate(dia = map(linearModel, \(f) {
    autoplot(f, smooth.colour = NA, alpha = 0.7, colour = "steelblue") +
      theme_bw(base_size = 8)
  } )) |>
  # Add the behaviour as a title to each diagnostic plot:
  mutate(dia = map2(dia, behaviour, \(dia, m) {
    gridExtra::grid.arrange(grobs = dia@plots, top = m)
  } )) |>
  # Suppress on-screen messages from the output:
  suppressMessages() |>
  capture.output() |>
  invisible()
```

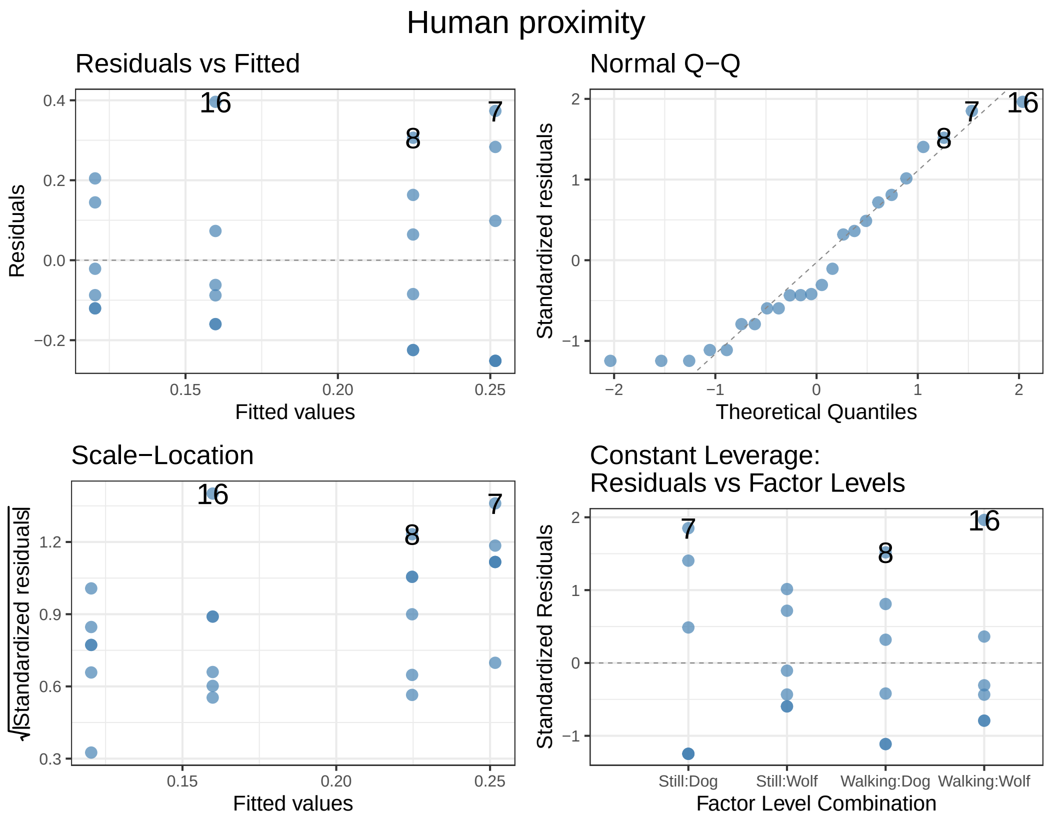

## Same direction

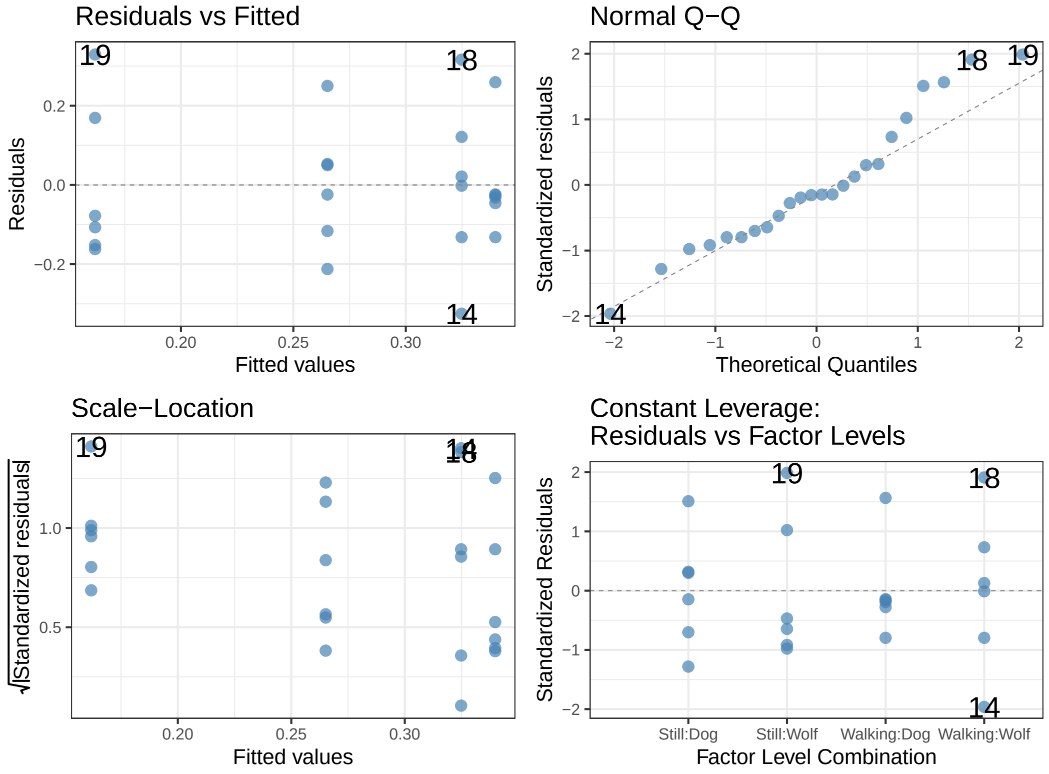


## Movement

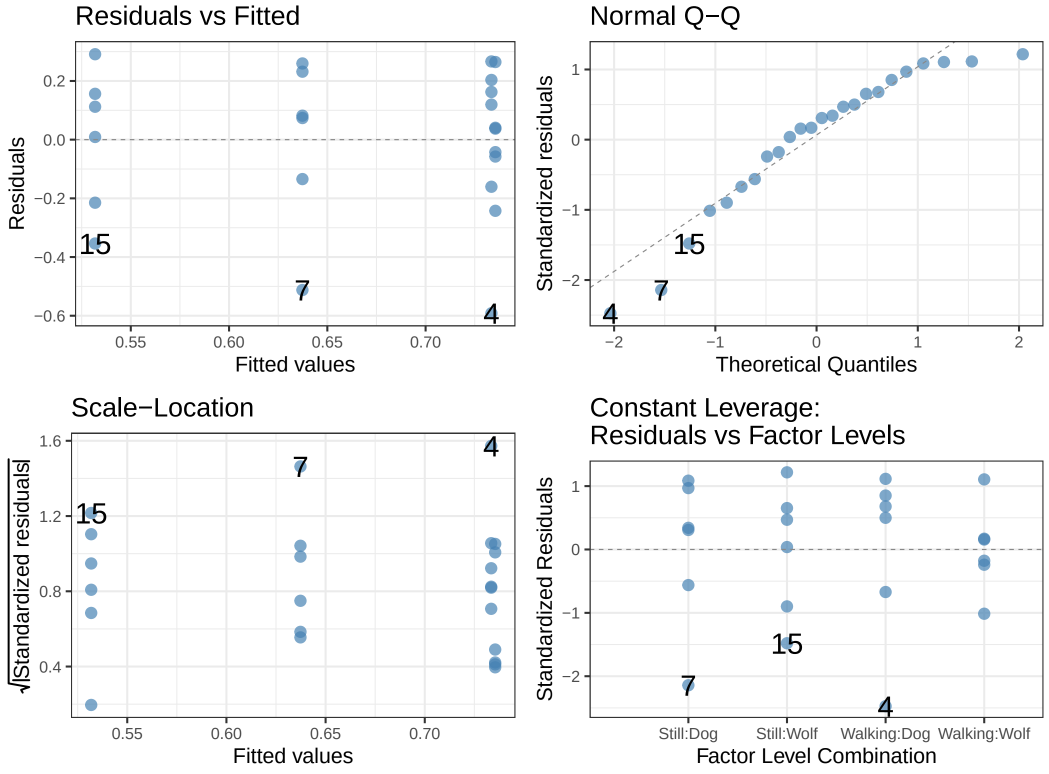

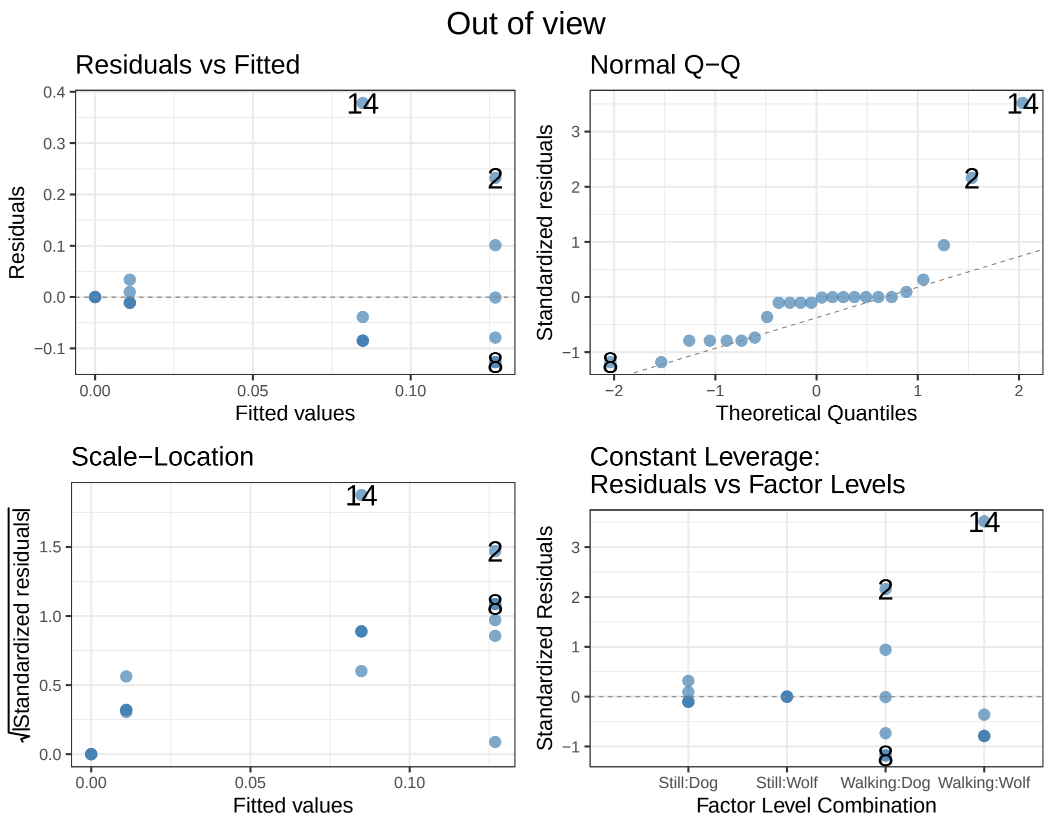


These plots look less than amazing. “Human proximity”, “Movement”, and “Same direction” are borderline, but “Out of view” is clearly unacceptable. As such, no results gleaned from the linear model should be taken as conclusive.

With that note of caution, here are the ANOVA tables. Just like in Section 1, here we again have a balanced and orthogonal design (6 observations per each unique combination of group and phase, for each behaviour), so type-I, type-II, and type-III sum of squares all yield the same results. We therefore default to type-I below:

```
regrDatC |>
  # Extract ANOVA table:
  mutate(linearModel = map(linearModel, compose(broom::tidy, anova))) |>
  # Reshape table to human-readable form:
  mutate(linearModel = map(linearModel, \(x) {
    select(x, term, p.value) |>
      drop_na()
  } )) |>
  unnest(linearModel) |>
  mutate(p.value = round(p.value, 4)) |>
  select(behaviour, term, `p-value` = p.value) |>
  # Easier visual detection of significant results:
  mutate(significance = ifelse(`p-value` < 0.05, "*", "")) |>
  knitr::kable()
```

| behaviour | term | p-value | significance |
|---|---|---|---|
| Human proximity | phase | 0.9455 | |
| Human proximity | group | 0.2899 | |
| Human proximity | phase:group | 0.7164 | |
| Same direction | phase | 0.1240 | |
| Same direction | group | 0.4334 | |
| Same direction | phase:group | 0.5574 | |
| Movement | phase | 0.1763 | |
| Movement | group | 0.6337 | |
| Movement | phase:group | 0.6208 | |
| Out of view | phase | 0.0500 | |
| Out of view | group | 0.5877 | |
| Out of view | phase:group | 0.7506 | |

The model fails to find evidence that any of these factors make a difference. The one possible exception is "Out of view", which might be influenced by phase ($p = 0.05$). Indeed, the suggested pattern is visible in Figure 4 as well.

The exact same conclusions obtain from the Scheirer–Ray–Hare test, which likely yields a better assessment of the differences than the linear model due to the small sample size and non-normal distribution of residuals apparent in the diagnostic plots:

```
regrDatC |>
  mutate(scheirerRayHare = map(scheirerRayHare, \(x) {
    mutate(x, term = rownames(x), .before = 1) |>
      filter(term != "Residuals")
  } )) |>
  unnest(scheirerRayHare) |>
  mutate(p.value = round(p.value, 4)) |>
  select(behaviour, term, `p-value` = p.value) |>
  mutate(significance = ifelse(`p-value` < 0.05, "*", "")) |>
  knitr::kable()
```

| behaviour | term | p-value | significance |
|---|---|---|---|
| Human proximity | phase | 0.8588 | |
| Human proximity | group | 0.4767 | |
| Human proximity | phase:group | 0.9527 | |
| Same direction | phase | 0.1938 | |
| Same direction | group | 0.7290 | |
| Same direction | phase:group | 0.4704 | |
| Movement | phase | 0.1488 | |
| Movement | group | 0.3122 | |
| Movement | phase:group | 0.7508 | |
| Out of view | phase | 0.0390 | * |
| Out of view | group | 0.1302 | |
| Out of view | phase:group | 0.7832 | |

# 4 Session Information

```r
sessionInfo()
```

```
R version 4.2.2 (2022-10-31)
Platform: x86_64-apple-darwin17.0 (64-bit)
Running under: macOS Big Sur ... 10.16

Matrix products: default
BLAS:   /Library/Frameworks/R.framework/Versions/4.2/Resources/lib/libRblas.0.dylib
LAPACK: /Library/Frameworks/R.framework/Versions/4.2/Resources/lib/libRlapack.dylib

locale:
[1] en_US.UTF-8/en_US.UTF-8/en_US.UTF-8/C/en_US.UTF-8/en_US.UTF-8

attached base packages:
[1] stats     graphics  grDevices utils     datasets  methods   base

other attached packages:
 [1] ggfortify_0.4.15 lubridate_1.9.2  forcats_1.0.0    stringr_1.5.1
 [5] dplyr_1.1.4      purrr_1.0.2      readr_2.1.4      tidyr_1.3.1
 [9] tibble_3.2.1     ggplot2_3.4.2    tidyverse_2.0.0

loaded via a namespace (and not attached):
 [1] nlme_3.1-160        matrixStats_0.62.0 bit64_4.0.5
 [4] httr_1.4.4          numDeriv_2016.8-1.1 TMB_1.9.6
 [7] tools_4.2.2         backports_1.4.1     utf8_1.2.4
[10] R6_2.5.1            nortest_1.0-4       DBI_1.1.3
[13] mgcv_1.8-41         colorspace_2.0-3    withr_3.0.0
[16] tidyselect_1.2.0    gridExtra_2.3       Exact_3.2
[19] emmeans_1.10.0      bit_4.0.5           compiler_4.2.2
[22] rcompanion_2.4.18   cli_3.6.2           expm_0.999-6
[25] sandwich_3.0-2      labeling_0.4.2      scales_1.2.1
[28] lmtest_0.9-40       mvtnorm_1.1-3       proxy_0.4-27
[31] multcompView_0.1-8  digest_0.6.30       minqa_1.2.5
[34] rmarkdown_2.18      pkgconfig_2.0.3     htmltools_0.5.7
[37] lme4_1.1-31         fastmap_1.1.0       highr_0.9
[40] rlang_1.1.3         readxl_1.4.2        rstudioapi_0.14
[43] farver_2.1.1        generics_0.1.3      zoo_1.8-11
[46] jsonlite_1.8.4      vroom_1.6.0         car_3.1-1
[49] magrittr_2.0.3      modeltools_0.2-23   Matrix_1.5-1
[52] Rcpp_1.0.12         DescTools_0.99.47   munsell_0.5.0
[55] fansi_1.0.6         abind_1.4-5         lifecycle_1.0.4
[58] stringi_1.8.3       multcomp_1.4-20     yaml_2.3.6
[61] carData_3.0-5       MASS_7.3-58.1       rootSolve_1.8.2.3
[64] plyr_1.8.8          grid_4.2.2          parallel_4.2.2
[67] crayon_1.5.2        lmom_2.9            lattice_0.20-45
[70] splines_4.2.2       hms_1.1.2           knitr_1.40
```

```
[73] pillar_1.9.0        boot_1.3-28         gld_2.6.6
[76] estimability_1.4.1  codetools_0.2-18    stats4_4.2.2
[79] glue_1.7.0          evaluate_0.18       data.table_1.14.4
[82] vctrs_0.6.5         nloptr_2.0.3        tzdb_0.4.0
[85] cellranger_1.1.0    gtable_0.3.1        xfun_0.41
[88] coin_1.4-2          xtable_1.8-4        libcoin_1.0-9
[91] broom_1.0.4         e1071_1.7-12        coda_0.19-4
[94] class_7.3-20        survival_3.4-0      glmmTMB_1.1.7
[97] timechange_0.1.1    TH.data_1.1-1       ellipsis_0.3.2
```
